# Social Collaboration Does Not Shape the Effects Caused by Self‐Encoding: Evidence From Ongoing and Enduring Collaboration

**DOI:** 10.1002/pchj.70006

**Published:** 2025-02-27

**Authors:** Aiqing Nie, Shuo Sun, Mingzheng Wu

**Affiliations:** ^1^ Department of Psychology, School of Education Science Shanxi Normal University Taiyuan Shanxi China; ^2^ Department of Psychology and Behavioral Sciences Zhejiang University Zhejiang Hangzhou China

**Keywords:** collaboration, emotional valence, episodic memory, self‐positivity bias, self‐reference

## Abstract

Past studies have illustrated that we tend to prioritize remembering information that is relevant to ourselves, resulting in a self‐reference effect. This effect is often influenced by emotions associated with the stimuli, frequently showcasing a self‐positivity bias. However, these effects have only been observed in individual memory, without any consideration given to a social collaboration setting. The current study intended to clarify these effects in ongoing and enduring social collaboration. Participants were instructed to encode personality trait adjectives, displayed in different colors with various emotional valences, using either self‐reference or other‐reference methods. They were then tasked with individually or collaboratively recalling the words along with their associated encoding task, followed by individual recall. Our data indicated evidence of the self‐reference effect in item memory during both ongoing and enduring collaborative sessions. This effect was evident for words studied in red, but the pattern was reversed for those in green. Additionally, the self‐positivity bias was observed when retrieving the source of the encoding task during ongoing collaborative sessions. A reversed self‐positivity bias was observed in item memory for words that were studied in green. An unexpected finding was that whether participants collaborated or not did not influence the effects we were investigating. Overall, we have extended the self‐reference effect and self‐positivity bias to the social collaboration setting, demonstrating that these effects remain consistent even in collaborative environments. This suggests that the underlying theories driving the effects are not contingent on social interaction. Moving forward, potential future directions for research are considered.

## 1 Introduction

In our daily lives, we often prioritize information that is relevant to ourselves, ultimately enhancing our memory retention. This phenomenon is known as the self‐reference effect. While several studies have explored the role of emotions in triggering the self‐reference effect, the evidence of its potential impact is inconsistent. Additionally, previous investigations have focused solely on individual memory, neglecting to examine whether the self‐reference effect also occurs in social collaboration. To fully grasp the self‐reference effect in a social collaboration setting, our goal is to address several unresolved issues and gain a comprehensive understanding.

To specify, the issues to be addressed include whether a reliable self‐reference effect occurs in a collaborative setting, and is the effect comparable to that in individual scenarios. Furthermore, it is crucial to investigate if the patterns of this effect vary across different memory tasks. Additionally, examining whether factors such as stimulus emotionality influence the effect is necessary. Furthermore, it is essential to determine if a significant self‐reference effect can be observed in a post‐collaboration test session for distinct memory tasks. Lastly, the study will explore whether a self‐positivity bias can be observed following social collaboration.

### 1.1 Paradigm Exploring the Self‐Reference Effect and Relevant Theoretical Accounts

It is widely acknowledged that we frequently process both information that is relevant to ourselves and information that is unrelated to us. To distinguish their similarities and differences in memory, researchers have developed a standardized research paradigm (e.g., Clarkson et al. 2022; Howell and Zelenski 2017; Kim et al. 2022; Nie, Zhou, et al. 2023; Rowell and Jaswal 2021; Yamawaki et al. 2017). Participants are expected to encode words describing personality traits by responding to various questions, such as “Does this word describe you?” or “Does this word describe person A?” These tasks are known as self‐encoding and other‐encoding tasks, respectively. The following test is to identify these studied words from a set of new words. Typically, individuals tend to remember self‐encoded words more effectively than those obtained through other‐encoding conditions. This benefit is commonly referred to as the self‐reference effect (Clarkson et al. 2022; Howell and Zelenski 2017; Kim et al. 2022; Rowell and Jaswal 2021; Yamawaki et al. 2017).

Several theoretical accounts have been proposed to explain the self‐reference effect. First, it is now recognized that individuals possess a self‐memory system, a conceptual framework that suggests the presence of a stable, long‐term self, and a working self (Kalenzaga and Jouhaud 2018). The long‐term self encompasses conceptual knowledge, including personal beliefs, and an autobiographical base built upon memories spanning various periods. On the other hand, the working self is a cognitive framework that adapts to various situations and contains a subset of self‐related details. It has been proposed that the working self plays a crucial role in self‐encoding and memory retrieval. Adhering to the principle of self‐coherence can help individuals determine which specifics to encode and which events to remember (Kalenzaga and Jouhaud 2018).

The second explanation is the self‐knowledge framework, which suggests that reflecting upon ourselves can activate existing self‐knowledge structures. These structures can be used to build upon and organize new self‐relevant information. This process ultimately enhances the encoding and storage of self‐related information, resulting in the self‐reference effect (Hutchison et al. 2021; Kim et al. 2022; Nie, Zhou, et al. 2023; Rowell and Jaswal 2021).

The third explanation is the automatic attentional account. According to this account, there are automatic attentional responses to self‐cues, ensuring that self‐relevant material is perceived more quickly and can capture and sustain attention more effectively. Consequently, the processing preference prompted by self‐cues tends to reinforce the self‐reference effect (Humphreys and Siu 2016; Hutchison et al. 2021; Rowell and Jaswal 2021; Sui and Humphreys 2017).

### 1.2 The Impact of Emotional Valence of Stimuli on the Self‐Reference Effect

Researchers have examined the self‐reference effect in various situations of episodic memory so far. Episodic memory refers to the ability to remember personal experiences or explicitly recall past events. It involves both item memory and source memory. Item memory pertains to the ability to recall the event, whereas source memory involves remembering the specific information about how, when, or where the event took place (Durbin et al. 2017; Minor and Herzmann 2019; Nie and Wu 2023; Nie, Zhou, et al. 2023; Pereira et al. 2022; Symeonidou and Kuhlmann 2022; Xiao and Nie 2023). Dual‐process theories propose that item recognition is more dependent on the rapid, automatic process of familiarity, while item recall and source memory rely more heavily on the deliberate, controlled process of recollection (Minor and Herzmann 2019; Nie, Zhou, et al. 2023; Symeonidou and Kuhlmann 2022; Ye et al. 2019; Zhou et al. 2020). A consistent self‐reference effect has been observed in both item memory (Arnaud et al. 2005; Grilli et al. 2018; Gutchess and Kensinger 2018) and source memory (Durbin et al. 2017; Nie, Zhou, et al. 2023).

Some studies specifically focus on the contribution of emotion to the self‐reference effect in episodic memory (Arnaud et al. 2005; Grilli et al. 2018; Gutchess and Kensinger 2018; Kalenzaga and Jouhaud 2018; Kim et al. 2022; Nie, Zhou, et al. 2023). On the one hand, the findings regarding item memory are not always consistent. For example, a study by Arnaud et al. (2005) revealed that positive words yield a more pronounced effect than negative words in free‐recall tasks, indicating a self‐positivity bias. However, this difference is not apparent in recognition tasks. These results suggest that the variation is attributed, at least in part, to the retrieval process. Additionally, a self‐positivity bias can also be observed in patients (Kalenzaga and Jouhaud 2018). On the contrary, studies conducted by Kim et al. (2022) on young adults, as well as on healthy older adults according to Grilli et al. (2018) have discovered that the self‐reference effect is not influenced by the emotional nature of the stimuli.

On the other hand, the self‐positivity bias is confirmed in source memory. For instance, Kim et al. (2022) discovered that the memory for contextual features associated with nouns was greater for self‐encoded positive information compared to self‐encoded negative information. This suggests that self‐encoding for negative information requires a higher cognitive load in comparison to positive cases. Furthermore, our team has discovered that only positive words show a self‐reference effect in source memory tasks, thus highlighting the presence of a self‐positivity bias (Nie, Zhou, et al. 2023).

In brief, the self‐reference effect is evident in both item memory and source memory and is often influenced by the emotional nature of stimuli, typically demonstrating a self‐positivity bias. Thus far, research on the self‐reference effect and the self‐positivity bias primarily concentrates on individual memory cases. It is not yet clear whether these effects would be evident in interpersonal interactions, such as social collaboration.

### 1.3 The Inhibition of Social Collaboration on Memory Performance

People can create and share memories. Collaborative memory research delves into the benefits and costs that arise from social interaction (Abel and Bäuml 2020; Grysman et al. 2020; Nie and Deng 2023; Nie and Guo 2023, 2024; Nie, Li, et al. 2023; Pepe et al. 2021; also see Marion and Thorley 2016, for a review). To determine whether social collaboration impacts our memory in a disparate manner compared to individual efforts, a standard paradigm has been established. In this paradigm, participants are initially instructed to encode the stimuli on their own. During the subsequent memory test, some participants are asked to independently recall the studied information, while others are involved in collaborative efforts (Hood et al. 2023; Nie and Guo 2023, 2024; Nie, Li, et al. 2023; Pepe et al. 2021; also see Marion and Thorley 2016, for a review).

To determine whether members in a collaborative situation are reaching their full potential, conducting a performance comparison between collaborative groups and virtual nominal groups is highly recommended. The member count remains consistent in both collaborative and nominal groups. A nominal group is comprised of individuals brought together pseudo‐randomly, and its performance is measured by the amount of nonredundant information retrieved by its members, representing the group's maximum potential. The comparison typically reveals that collaboration hinders individuals from reaching their full potential, as collaborative groups show lower accuracy (Hood et al. 2023; Nie and Guo 2024; Nie, Li, et al. 2023; Pepe et al. 2021; Rossi‐Arnaud et al. 2023; Saraiva et al. 2023; also see Marion and Thorley 2016, for a review).

One dominant explanation for the decrease in accuracy in collaborative groups is the Retrieval Strategy Disruption Hypothesis (RSDH). According to RSDH, each individual has a preexisting cognitive structure that typically helps them develop an idiosyncratic cognitive organization for study materials. During a collaborative test, a participant's idiosyncratic cognitive organization and preferred retrieval strategies can easily be disrupted by others who use different strategies. This can ultimately lead to a decrease in accuracy among collaborators (Basden et al. 1997; Guazzini et al. 2020; Maswood et al. 2022; Nie and Guo 2024; Nie, Ke, et al. 2023; Nie and Liu 2024; Rossi‐Arnaud et al. 2020; also see Marion and Thorley 2016, for a review).

It has been discovered that the emotional valence of stimuli can influence the detrimental impact on accuracy caused by collaboration. One of our experiments demonstrated that when words were deeply encoded, accuracy in item memory was significantly more compromised in both positive and negative words compared to neutral words in collaborative scenarios. When recalling the color source for words, negative words had a significantly more detrimental effect compared to both neutral and positive words (Ke et al. 2017, Experiment 1). This suggests that in collaborative situations, retrieval strategies for emotional words are more disrupted among collaborators. Additionally, the disruption of retrieval strategies affects both item memory and source memory, albeit it shows slightly different patterns between these two tasks. Furthermore, when transitioning to a shallow encoding task, the retention of emotional words was still more impaired in collaborative situations, but not in retrieving their sources (Ke et al. 2017, Experiment 2). Therefore, the impact of stimulus emotionality on triggering the detrimental aspect of collaborative memory is influenced by the encoding task.

From the above literature review, it can be concluded that both the roles of self‐reference and social collaboration have been explored in memory. Self‐reference focuses on the encoding of stimuli, typically resulting in the self‐reference effect during retrieval. Social collaboration involves the retrieval of information, either individually or collaboratively, often revealing the detrimental effects of collaboration as collaborators perform worse than nominal participants. Existing literature has only shown the self‐reference effect during individual retrieval, and the pattern may vary based on the emotional content of the stimuli, demonstrating a self‐positivity bias.

This leads us to question whether the self‐reference effect and self‐positivity bias would still be present during collaborative retrieval. To enhance the importance of this study, we have elevated our consideration and intention. This is because we may either recall our experiences on our own or share them with others who may have similar experiences, particularly those that are highly relevant to ourselves, such as those involving self‐reference encoding. One may be wondering which is better: individual retrieval or collaborative recall. The answer to this question can help people determine the most effective way to consolidate self‐encoded information.

The importance of the above topic prompts us to raise the following considerations. It is uncertain whether a reliable self‐reference effect will occur in a collaborative setting, and whether the effect will be comparable to that in nominal scenarios. It is also worth questioning whether the patterns of this effect vary between item memory and source memory. Furthermore, we raise the question of whether the effect is influenced by factors such as stimulus emotionality. Our first objective is to address these unresolved issues.

### 1.4 The Facilitation of Memory Performance Triggered by Collaborative Experience

Although ongoing social collaboration can elicit worse memory performance, it has also been demonstrated that collaboration can improve subsequent individual memory. A frequent observation is that individuals who have previously worked together tend to have better memory accuracy compared to those who have not. This implies that participants' information storage is reshaped by their previous experiences with social interpersonal remembering (Bärthel et al. 2017; Nie et al. 2019; Nie and Guo 2023; Nie, Ke, et al. 2023; Nie, Li, et al. 2023; also see Marion and Thorley 2016, for a review).

The RSDH has proposed four distinct mechanisms that are triggered by prior collaboration to facilitate subsequent memory: relearning information that is already known through collaborative retrieval, receiving cross‐cueing from the remembered contents of others, gaining re‐exposure to items that have been retrieved by partners, and correcting errors made by collaborative partners (Abel and Bäuml 2020; Nie and Liu 2024; also see Marion and Thorley 2016, for a review).

Our extensive investigations have uncovered that the facilitation of memory performance, brought about by prior collaborative experiences, can be observed in circumstances involving item memory (Nie and Deng 2023; Nie et al. 2019; Nie, Ke, et al. 2023; Nie, Li, et al. 2023). However, this pattern is only evident in a limited number of studies when it comes to source memory (e.g., Nie, Ke, et al. 2023). Despite this, we are interested in investigating whether a significant self‐reference effect can be observed in a post‐collaboration test session on these tasks. Additionally, we are interested in determining whether a self‐positivity bias can be observed after social collaboration. These concerns represent our second objective.

### 1.5 The Current Study and Our Main Hypotheses

Combining the above two objectives, the current study aims to explore the following issues. (a) Whether the self‐reference effect can be observed in a collaborative setting? (b) Whether there is a variation in the self‐reference effect between nominal and collaborative memory conditions? (c) Does the self‐reference effect show a self‐positivity bias? (d) Will social collaboration have a lasting impact on the manifestation of both the self‐reference effect and the self‐positivity bias? (e) Whether the patterns of these effects differ between item memory and source memory tasks? (f) Whether our target effects demonstrate reliable sensitivity to the interplay among the manipulated conditions?

To address these issues, we designed a three‐phase experiment consisting of one study and two recall sessions (Recall 1 and Recall 2). We used trait adjectives as stimuli, representing three different emotional valences—positive, neutral, and negative. These words were presented in either red or green and were assigned either self‐encoding or other‐encoding tasks. Free‐recall was conducted during both Recall 1 and Recall 2. Participants were required to retrieve both the studied words and their previously associated encoding tasks (self‐reference or other‐reference), referred to as item memory and source memory, respectively. Recall 1 was conducted either independently or collaboratively, while Recall 2 was always carried out individually.

In terms of item memory, we will analyze performance by assessing the proportions of correctly recalled words. In source memory, we considered the proportions of correct source identifications by focusing on those of the correctly recalled item, referred to as the conditional source–identification measure (CSIM), which mirrors the measurements used in published investigations (e.g., Bell et al. 2017; Li and Nie 2023; Nie et al. 2024).

In Recall 1, within the context of item memory, we first anticipated a noticeable self‐reference effect (Hypothesis 1). As a result, we have incorporated theories such as the working self, the self‐knowledge framework, and/or the automatic attentional account into our current social collaboration. Second, we predicted a self‐positivity bias (Hypothesis 2). Furthermore, we were able to observe both the self‐reference effect and the self‐positivity bias in both collaborative and nominal cases, with the effects being more prominent in nominal situations. This could be attributed to the RSDH, which proposes that collaboration may hinder memory retrieval (Guazzini et al. 2020; Maswood et al. 2022; Rossi‐Arnaud et al. 2020; also see Marion and Thorley 2016, for a review).

Third, it was expected that the self‐reference effect and self‐positivity bias would be weaker in source memory as opposed to item memory (Hypothesis 3). This is because dual‐process theories suggest that source memory requires greater involvement in the recollection process (Minor and Herzmann 2019; Nie, Zhou, et al. 2023; Symeonidou and Kuhlmann 2022; Ye et al. 2019; Zhou et al. 2020). Finally, our controlled variables significantly interacted with each other, impacting these two effects (Hypothesis 4).

When examining Recall 2 after collaboration, we had the following expectations. First, we expected to observe a reliable self‐reference effect in both item memory and source memory tasks (Hypothesis 5). If this is verified, it could suggest the involvement of the working self, the self‐knowledge framework, and/or the automatic attentional account in post‐collaboration. Second, we hypothesized that there would be a self‐positivity bias (Hypothesis 6). Third, we hypothesized that these two effects would be evident in participants from both previous nominal groups and collaborative groups (Hypothesis 7). Specifically, we expected to discover that these effects would be more pronounced among participants who had experienced collaboration compared to those who had not. Finally, our controlled variables significantly interacted with one another to influence these two effects (Hypothesis 8).

## 2 Method

Our experiment protocols listed below adhere to the guidelines established in the Helsinki Declaration. The study has been approved by the Science and Technology Ethics Committee of Shanxi Normal University. Pre‐registration for this experiment, along with access to the materials and data, is available at https://osf.io/ue8x3/?view_only=52108c24439a43ef8ab7b726f00214a6.

### 2.1 Design

The experiment utilized a mixed design incorporating factors including encoding task (self‐reference and other‐reference), group (collaborative and nominal), emotional valence (positive, neutral, and negative), recall phase (Recall 1 and Recall 2), memory task (item memory and source memory), and color (red and green). The group served as a between‐subject variable, while all other factors were within‐subject variables. The reference task, color, and emotional valence were associated with the words during the study phase. During both Recall 1 and Recall 2 phases, participants were directed to carry out tasks involving item memory and source memory, regardless of whether they were in nominal or collaborative groups. The encoding task served as the source.

### 2.2 Participants

We initially recruited a total of 108 participants for our experiment. They were randomly assigned to nominal and collaborative groups. During Recall 1, the collaborative groups consisted of 56 participants, of which 50 were females and 6 were males, with an average age of 20.20 years (SD = 1.93). These participants were paired into 28 collaborative groups, each comprising two members. Building on previous literature (Harris et al. 2019; Nie et al. 2024; Zhang et al. 2023), we ensured that individuals within the same group were strangers to one another before they arrived at our laboratory. Additionally, another 50 participants were randomly paired to form 25 nominal groups, comprising 41 females and 9 males, with a mean age of 20.76 years (SD = 2.46).

After excluding two outlier data points following Recall 1, we were able to retain valid data from 106 participants. This resulted in a total of 28 collaborative groups and 25 nominal groups. Upon conducting Recall 2, we removed data from three groups that did not meet the requirements, as well as excluding two outliers. Consequently, we retained valid data from 100 participants, with 50 belonging to collaborative groups and 50 to nominal groups.

All participants reported that they were right‐handed and had normal or corrected‐to‐normal vision. They stated that Chinese was their mother tongue and had no history of mental illness, neurological injury, and color blindness. They were selected to participate in exchange for either course credits or monetary compensation. All participants signed a written informed consent form before participating in the experiment. After completing the experiment, all participants received thanks and were provided with a debriefing.

To ensure an adequate sample size, we performed a sensitivity power analysis using G*Power v3.1 Software (Faul et al. 2009). When analyzing our current variables in a mixed analysis of variance (ANOVA) with a small‐to‐medium effect size (*f* = 0.21), and assuming a two‐tailed *α* = 0.05 and 1 − *β* = 0.80, a minimum of 40 groups was determined to be necessary. These groups would be divided into 20 collaborative and 20 nominal groups. Therefore, our group size exceeded the necessary threshold.

### 2.3 Materials

We selected 168 personality trait adjectives with varying emotions to serve as the foundation for our formal stimuli. The adjectives were easy to read and had clear meanings. The adjectives were sourced in two ways. First, we identified 56 positive adjectives, 56 negative adjectives, and 34 neutral adjectives from existing literature (Van Den Joke et al. 2014; Roca et al. 2023). Second, we augmented our list by incorporating an additional 22 neutral adjectives from the database of the Chinese Affective Words System (CAWS) (Wang et al. 2008). Therefore, the adjectives were evenly distributed among the positive, neutral, and negative categories, with a total of 56 words in each. They were then prepared in Chinese to match the mother tongue of our current participants. Most of the words are typically comprised of two characters.

Afterwards, we invited 31 volunteers, who displayed similar characteristics to our formal participants but did not participate in the formal experiment, to evaluate the emotional valence of the words. The evaluation was made on a nine‐point Likert scale. A rating of 9 indicated extreme positivity, 5 denoted neutrality, and 1 represented extreme negativity. This method bears a resemblance to the approach used in prior literature (Montefinese et al. 2014; Warriner et al. 2013).

Based on this assessment, 144 words were selected as formal experimental stimuli. Each emotional valence was represented by 48 words. Specifically, words scoring below 3.5 (*M* = 3.006, SE = 0.032) were categorized as negative, those scoring between 3.8 and 6.2 (*M* = 4.902, SE = 0.097) were categorized as neutral, and any word scoring above 6.5 (*M* = 7.376, SE = 0.045) was categorized as positive. This classification followed previous literature (Van Den Joke et al. 2014; Nie, Zhou, et al. 2023; Roca et al. 2023).

A one‐way ANOVA confirmed significant differences among the three emotional valences, *F*(2,165) = 806.524, *p* < 0.001, $\eta_{p}^{2}=0.907$. Specifically, subsequent pairwise *t*‐tests indicated a substantial difference between positive and negative valences, as indicated by *t*(55) = 90.201, *p* < 0.001, Cohen's *d* = 12.052, 95% confidence interval (CI) (4.273, 4.467). Similarly, a clear distinction was identified between positive and neutral valences, as reflected by *t*(55) = 24.348, *p* < 0.001, Cohen's *d* = 3.255, 95% CI (2.271, 2.678). Additionally, neutral and negative valences also showed significant differences, as evidenced by *t*(55) = 18.946, *p* < 0.001, Cohen's *d* = 2.531, 95% CI (−2.096, −1.695).

Aside from the formal stimuli, the remaining 24 words were used as practice trials. The practice trials were conducted to ensure that participants understood the task instructions and were familiar with the experimental procedure. The words were evenly distributed among various encoding tasks and emotional valences in both the practice and formal experiments. To reduce interference among stimuli and minimize the memory load on participants, we divided the formal experimental words into four blocks. Each block consisted of 36 words, with half exposed to self‐reference encoding and the other half being exposed to self‐reference encoding. Therefore, six words per emotional valence were provided for both encoding cases per block. Furthermore, we ensured an even distribution of color display (red or green) among the encoding tasks and emotional valences for words.

### 2.4 Procedure

**FIGURE 1:**
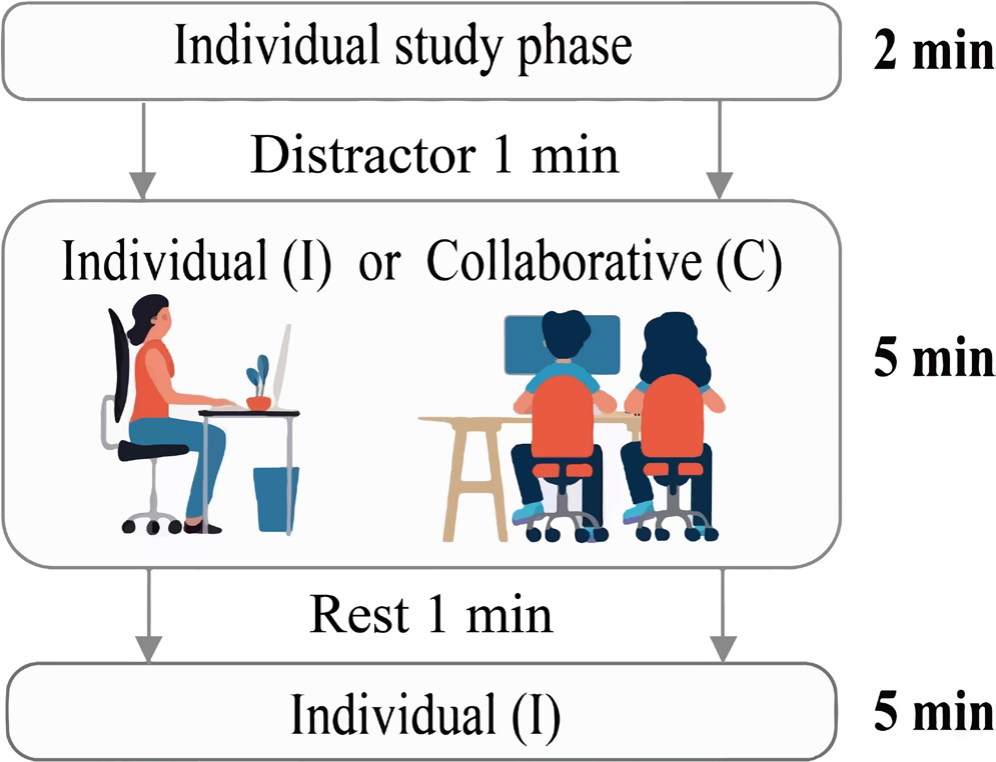
Experimental procedures and the time required for each phase of one study‐test block.

The entire experiment lasted approximately 70 min. After completing the practice trials, participants proceeded to engage in the four formal blocks. The procedures for both the practice and the formal experiment were consistent and consisted of three core sessions: study, Recall 1, and Recall 2, along with the distractions (see Figure 1).

#### 2.4.1 Study Session

The participants were instructed to complete the study task, with the understanding that they would later be assigned either individual or collaborative recalls. Throughout the study session, participants were seated before a table in a comfortable position and were engaged in the tasks independently. The members of the nominal groups entered the lab individually, ensuring that only one participant was enrolled at a time. In a collaborative setting, two participants per group were stationed in adjacent rooms to prevent interference from each other, while they were instructed to begin each study task simultaneously.

Participants were first presented with task instructions outlining two distinct tasks to be performed: self‐reference and other‐reference tasks. In the self‐reference task indicated by “自我参照,” participants were asked to evaluate how they would be perceived by others as either desirable, neutral, or undesirable based on the displayed trait adjectives by pressing the corresponding keys on the keyboard: “F” for desirable, “B” for neutral, and “J” for undesirable. The other‐reference task was prompted by the concept of “他人参照,” which guided participants to determine whether “a person” would be considered desirable, neutral, or undesirable if they possessed the trait represented by a word. Additionally, participants were asked to indicate their feelings by pressing the corresponding keys: “F” for desirable, “B” for neutral, and “J” for undesirable.

Participants were instructed to complete the key presses during the presentation interval of a word and its subsequent inter‐stimulus interval (ISI). They were encouraged to make quick and accurate judgments. Additionally, participants were informed that the adjectives would be presented in red or green. They were instructed to remember each adjective along with its displayed color and encoding task, as this information would be crucial for the tests ahead. To mitigate contamination caused by the task order, the two encoding tasks were counterbalanced between blocks.

There was first a white fixation cross, lasting 1300 ms, that appeared at the center of the screen to direct participants' attention to where the word would appear. The fixation cross was followed by a word displayed for 1200 ms, either in red or green. Afterwards, a 1300 ms ISI occurred with a white fixation cross displayed at the center of the screen before the next trial began. All fixation crosses and words were shown against a solid black background. To maintain variety and prevent predictability, words were displayed in a pseudo‐random order to ensure that no three consecutive occurrences had the same emotional valence or color.

**FIGURE 2:**
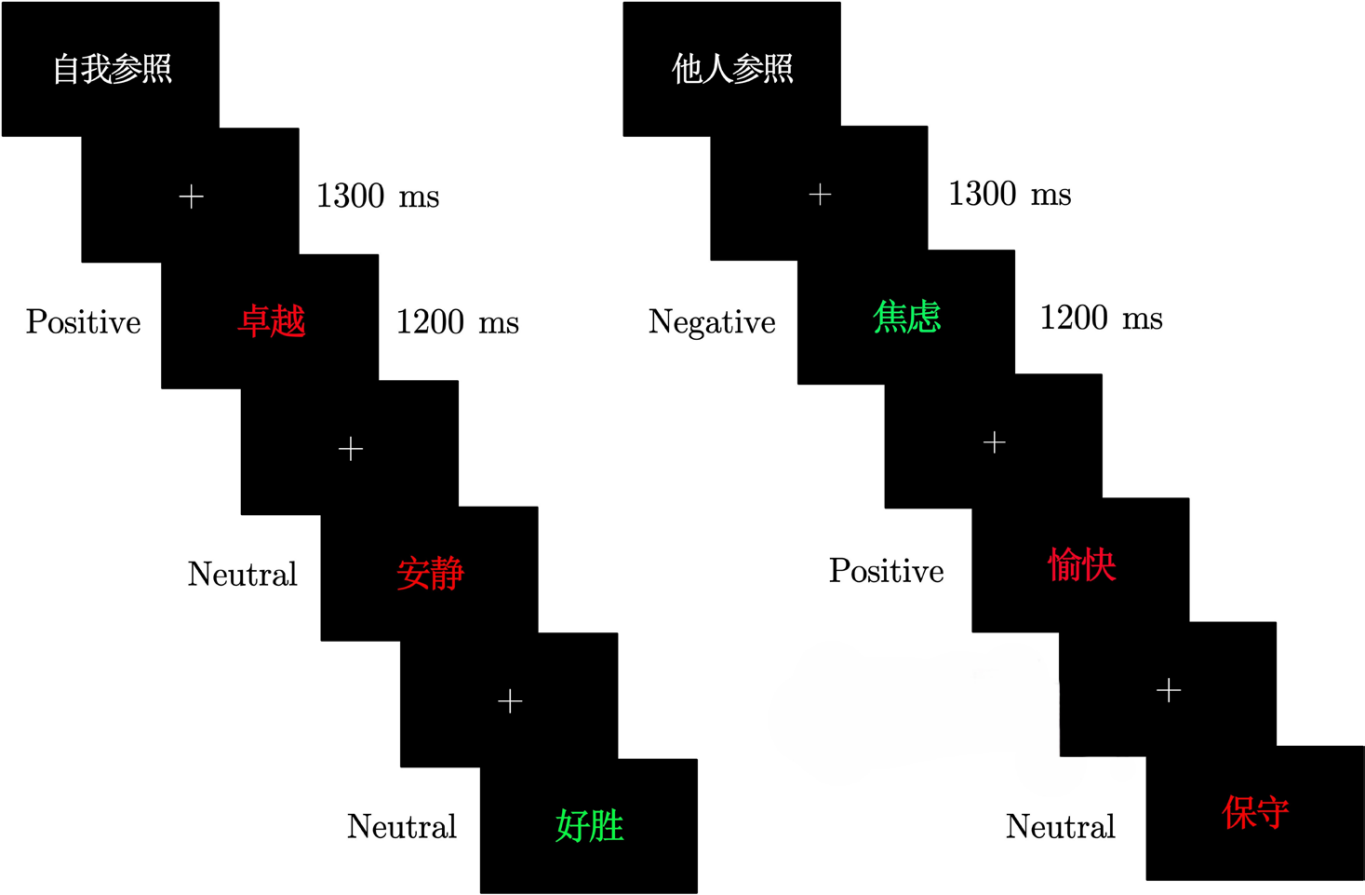
Schematic representation of the study phase and stimulus exemplars. “自我参照” refers to the self‐reference task, with “卓越,” “安静,” “好胜” meaning “Excellent,” “Quiet,” and “Competitive,” respectively. “他人参照” refers to the other‐reference task, with “焦虑,” “愉快,” “保守” meaning “Anxiety,” “Happiness,” and “Conservative,” respectively.

The stimuli were programmed using the E‐Prime 2.0 Software created by Psychology Software Tools, INC. Each study block lasted approximately 2 min. All words were displayed in the bold Microsoft Ya Hei font, with a font size of 150. The computer screen had a resolution of 1920 × 1080 pixels and a refresh rate of 100 Hz. Participants were seated approximately 60 cm from the screen center and were instructed to focus their gaze at a height equal to the center of the screen. Figure 2 provides a schematic representation of the study phase and stimulus exemplars.

#### 2.4.2 Distraction

To prevent participants from rehearsing the words they had just studied, a 1‐min distraction occurred following the study session. The distraction was conducted in the same room as for the study phase. The distraction involved completing simple arithmetic problems, like solving equations such as “48 − 33 = ?”. Each participant was instructed to record their answers individually on an answer sheet. Once the 1‐min interval marked by a timer had elapsed, recall tests commenced, regardless of how many arithmetic problems were solved.

#### 2.4.3 Test Sessions

The test sessions consisted of Recall 1 and Recall 2. Both included item memory and source memory tasks. To comprehensively assess participants' memory retrieval processes, the tasks were skillfully conducted using the sequential paradigm, as controlled by previous publications (Nie et al. 2023, 2024; Xiao and Nie 2023). Participants first completed an item memory test to recall the studied words, followed by a source memory task. The nominal participants consistently recalled information on their own. However, the collaborative dyads were first recalled collaboratively in Recall 1 and then recalled individually in Recall 2.

#### 2.4.4 Recall 1

During the Recall 1 phase, participants were briefed on their involvement with item memory and source memory tasks. They were allocated a 5‐min epoch to perform the tasks for each block. The allocation of a 5‐min interval was determined by the results of a pilot study, which showed that nearly both the nominal and collaborative groups were able to complete the tasks within 3–4 min. At the end of the 5‐min interval, participants were instructed to stop their recall activities.

The nominal participants remained in their rooms and completed the Recall 1 tasks individually. The data from this phase were collected using a program generated by E‐Prime 2.0. During the program, the screen initially displayed a black fixation cross for 500 ms, followed by white text‐box cycles to enter answers. The first step was a text box that allowed participants to enter a word. When participants input a recalled word, they were then directed to the source memory task. Participants were strongly encouraged to type Chinese characters corresponding to each word. They could type the words in any order they preferred. The source task had two options, where “1” represented “Self‐reference” and “2” indicated “Other‐reference.” Although these tasks had no time limits, participants were asked to prioritize speed and accuracy.

Except for the members involved at any given time, the method of inputting answers was the same between nominal and collaborative groups. To facilitate collaboration, one member from each group was directed to enter their partner's room and work together in front of a single computer screen. The two members per group were instructed to collaborate in a free‐flowing procedure, as outlined in previous literature (Nie and Guo 2024; Nie, Ke, et al. 2023; also see the meta‐analysis in Marion and Thorley 2016). This procedure allowed collaborators to make responses and address any discrepancies freely. One member was responsible for recording the answers using the E‐Prime program as the nominal participants did, while the recording roles of the members were rotated across different blocks.

#### 2.4.5 Rest

After completing Recall 1, individuals in the nominal groups continued to stay in their assigned rooms. However, one member from each collaborative group should return to their original study room. To prevent potential fatigue, each participant was then given a 1‐min break to rest. After this short break, Recall 2 began.

#### 2.4.6 Recall 2

During this phase, all participants were seated in separate rooms and performed a second free‐recall independently. The recall interval, test arrangements, and answer input were kept consistent with those for the nominal participants during Recall 1.

#### 2.4.7 Breaks Between Two Consecutive Blocks

To mitigate recency effects and inter‐block interference, as well as to reduce participants' memory load, a 3‐min rest break was provided after the completion of a block. Subsequently, the next block began once the break had concluded.

### 2.5 Coding for the Recalled Answers

Our primary focus was on the responses provided by participants for both Recall 1 and Recall 2, which included both item memory and source memory tasks. Therefore, only the entered answers were taken into consideration. Separate coding was applied for Recall 1 and Recall 2 to determine correct answers.

It is worth noting that Recall 1 focused on group data collected from two members per group. Due to the collaborative nature of Recall 1, each group had one answer set that provided information on the group's performance. The data for each nominal group were taken from the answer sets of its two members, allowing us to gather a pool of nonredundant recalled information. As mentioned in the Introduction, the above method has been widely utilized (Greeley et al. 2024; Hood et al. 2023; Montoro‐Membila et al. 2022; Nie et al. 2019, 2024; Nie and Li 2021, 2024; Nie, Li, et al. 2023; Saraiva et al. 2023). Furthermore, in instances where one group member accurately recalled information while another did not, the collective response was considered accurate. This approach was applied to both item memory and source memory tasks. The data collected for Recall 2 was gathered from individual participants in both nominal and collaborative groups.

The answers were coded separately for item memory and source memory tasks. For the item memory task, a rigorous criterion was employed to minimize the influence of guesses. Only responses that were exact replicas of the items previously studied were considered correct. For instance, if a studied item like “好胜” (reads “competitive”) was inputted as its sound‐alike, such as “好生,” “号胜,” or “毫升,” they were marked as errors. This method reflects the manipulations employed in prior research (Harris et al. 2022; Nie and Guo 2023). Through this analysis, we determined the proportions of correctly recalled words. Accurate performance in source memory included data related to both self‐reference and other reference. Since we used the sequential paradigm, the data for source memory were founded on item memory. Specifically, we measured the CSIM rates as employed in published investigations (e.g., Bell et al. 2017; Li and Nie 2023; Nie et al. 2024).

Our task involved a recall activity where participants typed their answers. However, this method may have inadvertently missed some correct answers. The following cases may have occurred: (i) participants knew the correct answer but mistakenly typed it; (ii) participants knew the correct answer but, because they inputted other answers first, they ended up forgetting to enter this particular answer; (iii) participants who were familiar with the structure of a word but forgot how to pronounce it may have chosen to use the pinyin input method instead of the other input method. In these instances, they may have decided not to input the answer at all. Furthermore, these cases may occur in both nominal and collaborative participants. While these situations may have occurred, we cannot determine what happened in the participants' minds or what they experienced while entering their answers. Therefore, we had to rely solely on their input responses for our data analysis. This method was balanced between the nominal and collaborative participants, ensuring that the above cases were evenly distributed between the two groups and that our data pattern would not be affected.

## 3 Results

We separately calculated the correct recall proportions for nominal and collaborative groups in both item memory and source memory tasks. Also, we conducted separate analyses for the data obtained from Recall 1 and Recall 2. One of the reasons for conducting these separate analyses was to ensure consistency with previous studies that involved manipulating multiple test sessions (Bärthel et al. 2017; Nie et al. 2021, 2024; Nie, Ke, et al. 2023; Nie, Li, et al. 2023). Another reason for this discrepancy was the inconsistency in sample size between the two recall sessions. Recall 1 involved group recall, while Recall 2 involved individual recall. Furthermore, we analyzed the data obtained from item memory and source memory separately. This approach was necessary because the sequential paradigm made the outcomes of source memory depend on the performance of item memory.

Data analysis was conducted applying JASP (version 0.17.1.0) (JASP Team 2023), with all inferential statistical analyses set at a significance threshold of *p* < 0.05. The analysis employed a two‐tailed test. Assumptions were assessed before conducting a mixed ANOVA to ensure that our data met the assumption of homogeneity of variance. In cases where sphericity was violated, the degrees of freedom were adjusted using the Greenhouse–Geisser correction method. Each *F*‐test was reported including the *F*‐ratio, corrected *p*‐value, and effect size ($\eta_{p}^{2}$). Post hoc tests were conducted using the Bonferroni method, which considers multiple comparisons. The results were presented in the form of *t*‐values, *p*‐values, effect sizes measured by Cohen's *d*, and 95% CIs for mean differences. As our main focus was on the data related to the variable of the encoding task, we were interested in the self‐reference effect and the self‐positivity bias.

### 3.1 Results in Recall 1

#### 3.1.1 The Effects of Item Memory During Recall 1

**FIGURE 3:**
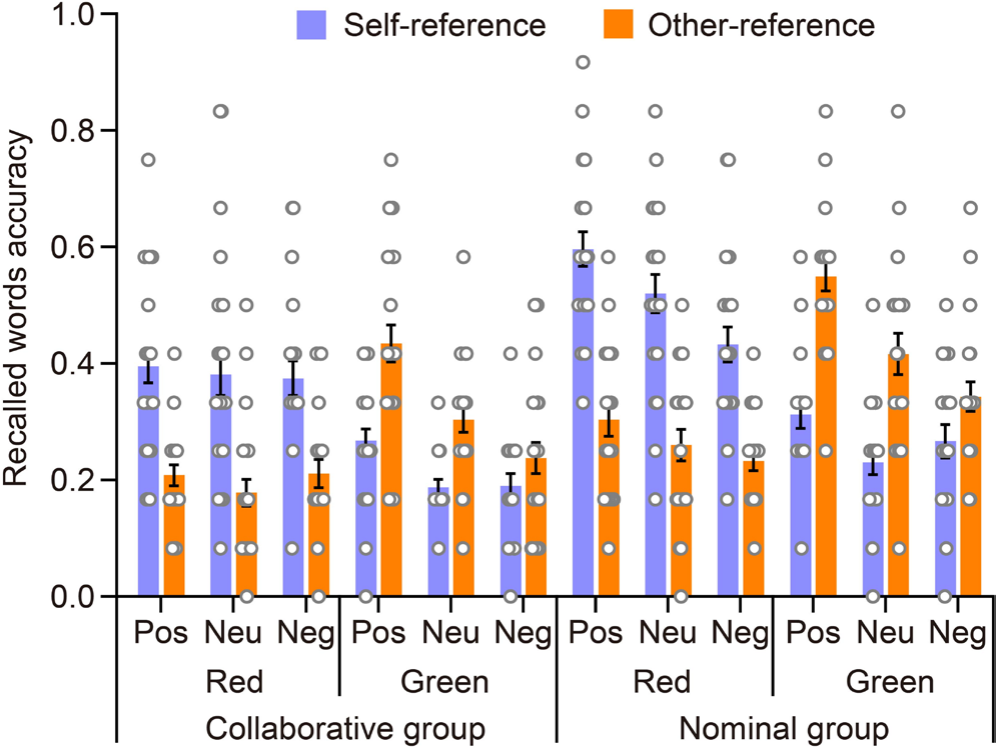
The accuracy of correctly recalled words in item memory during Recall 1, demonstrating the impact of the group by encoding task by emotional valence by color. The scattered dots depict the data for each group, with error bars representing standard errors. Pos, positive; Neu, Neutral; Neg, negative.

Figure 3 illustrates the accuracy of item memory during Recall 1. The figure is plotted as a function of the encoding task, categorized by group, color, and emotional valence. Accuracy for item memory of Recall 1 can also be found in Table 1. To explore the significance of the self‐reference effect in item memory of Recall 1, a mixed four‐way ANOVA was conducted. The analysis considered accuracy based on the variables of encoding task, group, color, and emotional valence.

**TABLE 1:** Accuracy (*M* and SE) for item memory of Recall 1, showing the function of color by encoding task by emotional valence by the group.

Color	Encoding task	Emotional valence	Group	*M*	SE
Red	Self‐reference	Positive	Collaborative	0.396	0.029
			Nominal	0.597	0.030
		Neutral	Collaborative	0.381	0.035
			Nominal	0.520	0.033
		Negative	Collaborative	0.375	0.029
			Nominal	0.433	0.030
	Other reference	Positive	Collaborative	0.208	0.018
			Nominal	0.303	0.028
		Neutral	Collaborative	0.178	0.023
			Nominal	0.260	0.027
		Negative	Collaborative	0.211	0.024
			Nominal	0.233	0.017
Green	Self‐reference	Positive	Collaborative	0.268	0.020
			Nominal	0.313	0.025
		Neutral	Collaborative	0.188	0.013
			Nominal	0.230	0.021
		Negative	Collaborative	0.190	0.021
			Nominal	0.267	0.028
	Other reference	Positive	Collaborative	0.435	0.031
			Nominal	0.550	0.025
		Neutral	Collaborative	0.304	0.021
			Nominal	0.417	0.036
		Negative	Collaborative	0.238	0.027
			Nominal	0.343	0.026

The ANOVA revealed a significant main effect of the encoding task, as evidenced by *F*(1,51) = 13.617, *p* < 0.001, $\eta_{p}^{2}=0.211$. The accuracy of the self‐reference case (*M* = 0.344, SE = 0.010) was significantly higher compared to that of the other‐reference case (*M* = 0.304, SE = 0.009), indicating evidence of the self‐reference effect. Furthermore, the encoding task did not interact with the group, as *F*(1,51) = 0.054, *p* = 0.817, $\eta_{p}^{2}=0.001$, indicating that the self‐reference effect was not affected by whether the participants collaborated or not. Notably, the encoding task significantly interacted with color, as indicated by *F*(1,51) = 400.375, *p* < 0.001, $\eta_{p}^{2}=0.887$. Post hoc tests showed that for words studied in red, there was a significant self‐reference effect, *t*(51) = 15.597, *p* < 0.001, Cohen's *d* = 1.617, 95% CI (0.180, 0.255). In contrast, for words studied in green, there was an opposite trend, *t*(51) = −9.910, *p* < 0.001, Cohen's *d* = −1.027, 95% CI (−0.176, −0.101).

Furthermore, the three‐way interaction between encoding task, color, and emotional valence was found to be significant, with an *F*(2,102) = 7.182, *p* = 0.001, $\eta_{p}^{2}=0.124$. Specifically, the words studied in red for all three emotional valences showed the self‐reference effect, with positive, neutral, and negative words showing the effects respectively: *t*(51) = 9.886, *p* < 0.001, Cohen's *d* = 1.784, 95% CI (0.158, 0.323); *t*(51) = 9.511, *p* < 0.001, Cohen's *d* = 1.717, 95% CI (0.148, 0.314); as well as *t*(51) = 7.476, *p* < 0.001, Cohen's *d* = 1.349, 95% CI (0.099, 0.264). For the words studied in green, the pattern of positive and neutral cases was reversed, with significant differences found in both cases: *t*(51) = −8.297, *p* < 0.001, Cohen's *d* = −1.497, 95% CI (−0.284, −0.119), and *t*(51) = −6.223, *p* < 0.001, Cohen's *d* = −1.123, 95% CI (−0.234, −0.069). However, there was no significant difference in the negative case, *t*(51) = −2.555, *p* = 0.734, Cohen's *d* = −0.461, 95% CI (−0.145, 0.021). Additionally, the four‐way interaction among encoding task, group, color, and emotional valence was not statistically significant, as indicated by *F*(2,102) = 0.533, *p* = 0.588, $\eta_{p}^{2}=0.010$.

Therefore, in terms of the item memory of Recall 1, we observed evidence of the self‐reference effect, which was not affected by whether participants collaborated or not. When the words were studied in red, a significant self‐reference effect was observed for positive, neutral, and negative words. However, for both positive and neutral words studied in green, the pattern was reversed.

**FIGURE 4:**
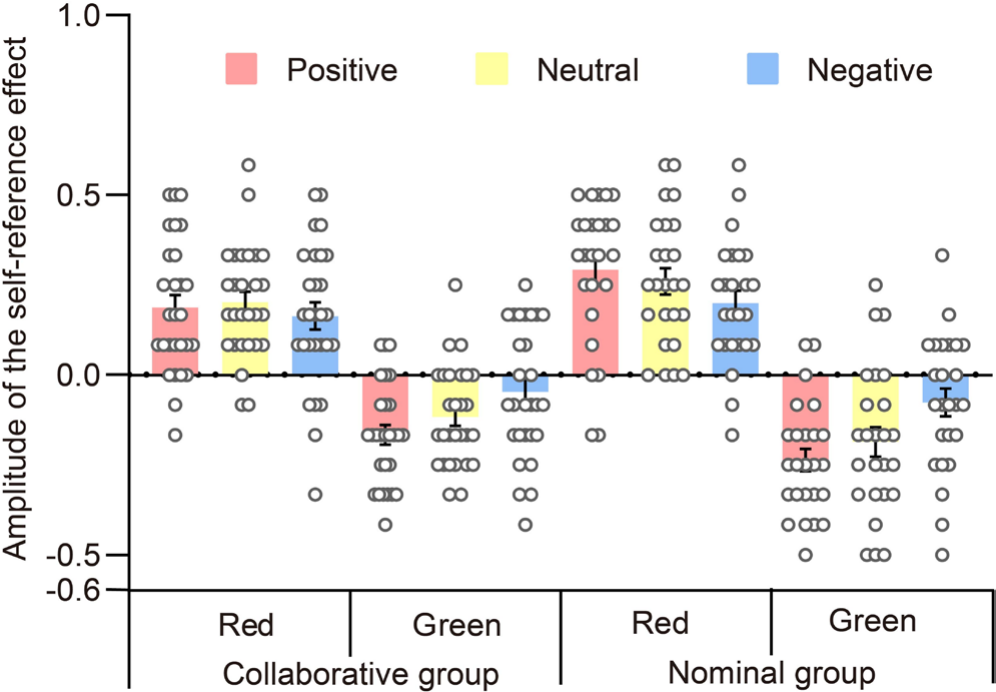
The amplitude of the self‐reference effect in Recall 1 is displayed by group, color, and emotional valence. Each scattered dot represents the data for every group, with error bars indicating standard errors.

Due to the influence of the emotional valence of words on the self‐reference effect, we conducted an examination of the magnitude of this effect (the accuracy of self‐reference compared to other‐reference) to verify the presence of a self‐positivity bias. The mixed ANOVA included group, color, and emotional valence as variables. Refer to Figure 4 for details on the magnitude of this effect.

The ANOVA did not confirm the main effect of emotional valence, *F*(2,102) = 1.752, *p* = 0.179, $\eta_{p}^{2}=0.004$, nor did the interaction between emotional valence and group, *F*(2,102) = 0.160, *p* = 0.852, $\eta_{p}^{2}=3.847\times 10^{-4}$. However, emotional valence interacted with color, *F*(2,102) = 7.183, *p* = 0.001, $\eta_{p}^{2}=0.027$. Post hoc tests revealed that for words studied in green, the amplitude of the self‐reference effect was significantly higher for positive words compared to negative words, *t*(51) = −4.050, *p* = 0.001, Cohen's *d* = −0.790, 95% CI (−0.242, −0.037). However, positive and neutral words did not differ from each other, nor did neutral and negative words, as indicated by the respective values of *t*(51) = −1.461, *p* = 1.000, Cohen's *d* = −0.285, 95% CI (−0.153, 0.052), and *t*(51) = −2.589, *p* = 0.155, Cohen's *d* = −0.505, 95% CI (−0.192, 0.013). For words studied in red, the amplitude of the self‐reference effect was similar among the three emotional valences, *p* = 1.000. Additionally, the three‐way interaction between color, emotional valence, and group was found not to be significant, *F*(2,102) = 0.533, *p* = 0.589, $\eta_{p}^{2}=0.002$. In conclusion, during the item memory of Recall 1, a reversed self‐positivity bias was observed for words studied in green, regardless of social collaboration.

#### 3.1.2 The Effects of Retrieving Sources During Recall 1

**FIGURE 5:**
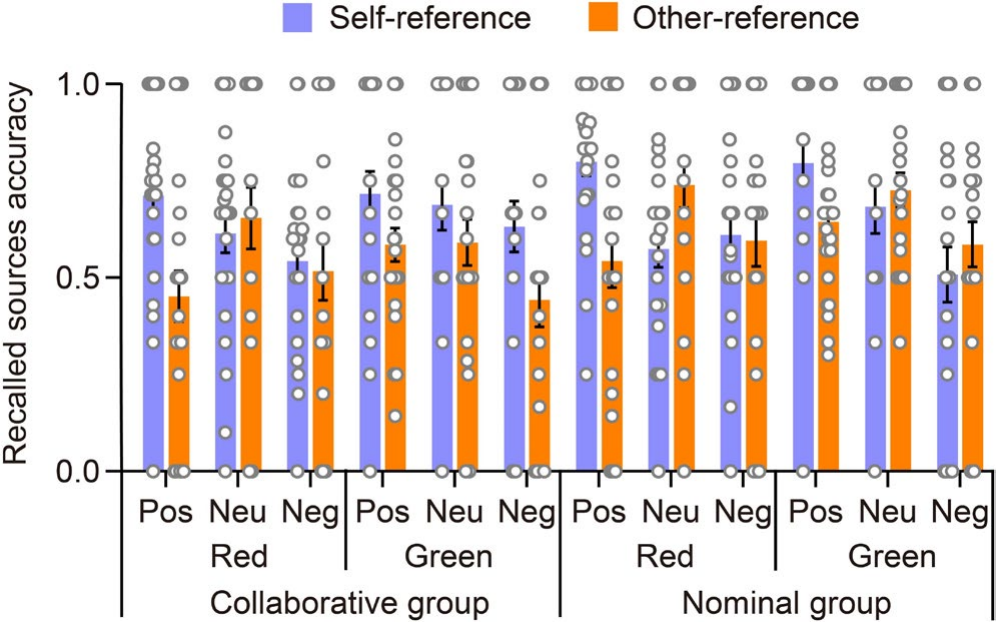
The CSIM rates for retrieving sources during Recall 1. The figure displays the data as a function of the encoding task, categorized by group, color, and emotional valence. The scattered dots represent the data for each group, with error bars denoting standard errors. Pos, positive; Neu, neutral; Neg, negative.

Figure 5 illustrates the CSIM rates in retrieving sources during Recall 1. The figure categorizes the data based on the encoding task, categorized by group, color, and emotional valence. To further explore the significance of the self‐reference effect in retrieving sources during Recall 1, the CSIM rates were analyzed using a mixed four‐way ANOVA, similar to the analysis conducted for the item memory task. CSIM rates for source memory of Recall 1 can also be found in Table 2.

**TABLE 2:** CSIM rates (*M* and SE) for source memory of Recall 1, showing the function of color by encoding task by emotional valence by the group.

Color	Encoding task	Emotional valence	Group	*M*	SE
Red	Self‐reference	Positive	Collaborative	0.712	0.046
			Nominal	0.799	0.037
		Neutral	Collaborative	0.615	0.050
			Nominal	0.575	0.048
		Negative	Collaborative	0.543	0.045
			Nominal	0.611	0.053
	Other reference	Positive	Collaborative	0.452	0.066
			Nominal	0.544	0.069
		Neutral	Collaborative	0.654	0.079
			Nominal	0.739	0.059
		Negative	Collaborative	0.517	0.075
			Nominal	0.595	0.066
Green	Self‐reference	Positive	Collaborative	0.717	0.057
			Nominal	0.795	0.052
		Neutral	Collaborative	0.688	0.065
			Nominal	0.683	0.069
		Negative	Collaborative	0.632	0.066
			Nominal	0.508	0.071
	Other reference	Positive	Collaborative	0.585	0.043
			Nominal	0.644	0.038
		Neutral	Collaborative	0.591	0.060
			Nominal	0.726	0.044
		Negative	Collaborative	0.443	0.069
			Nominal	0.586	0.058

The ANOVA confirmed a significant main effect of the encoding task, *F*(1,51) = 4.971, *p* = 0.030, $\eta_{p}^{2}$ = 0.089. Specifically, the CSIM rates for the self‐reference case (*M* = 0.656, SE = 0.017) were superior to the other‐reference case (*M* = 0.587, SE = 0.018), demonstrating evidence of the self‐reference effect. The encoding task did not interact with group, *F*(1,51) = 2.149, *p* = 0.149, $\eta_{p}^{2}$ = 0.040; nor did the encoding task interact with color, *F*(1,51) = 0.128, *p* = 0.722, $\eta_{p}^{2}=0.003$. The encoding task, however, did interact with emotional valence, *F*(1.934, 98.623) = 8.613, *p* < 0.001, $\eta_{p}^{2}=0.144$. Further post hoc tests for the reliable interaction revealed a significant self‐reference effect for positive words, *t*(51) = 4.427, *p* < 0.001, Cohen's *d* = 0.655, 95% CI (0.065, 0.334). However, no evidence of a significant self‐reference effect for neutral and negative words, *t*(51) = −0.833, *p* = 1.000, Cohen's *d* = −0.123, 95% CI (−0.172, 0.097), and *t*(51) = 0.851, *p* = 1.000, Cohen's *d* = 0.126, 95% CI (−0.096, 0.173).

Additionally, none of the other interactions were found to be significant. The three‐way interaction involving encoding task, group, and emotional valence yielded an *F*(2,102) = 0.997, *p* = 0.373, $\eta_{p}^{2}=0.019$. The interaction among encoding task, color, and emotional valence yielded an *F*(2,102) = 1.921, *p* = 0.152, $\eta_{p}^{2}=0.036$. The four‐way interaction among encoding task, group, color, and emotional valence did not reach statistical significance, *F*(2,102) = 0.721, *p* = 0.489, $\eta_{p}^{2}=0.014$.

The data above indicate that there was a reliable self‐reference effect for the source memory of Recall 1, particularly significant in positive words but not in neutral and negative words. This suggests a self‐positivity bias. Furthermore, these effects are not impacted by whether participants collaborated or not.

### 3.2 Results in Recall 2

#### 3.2.1 The Effects on Item Memory During Recall 2

**FIGURE 6:**
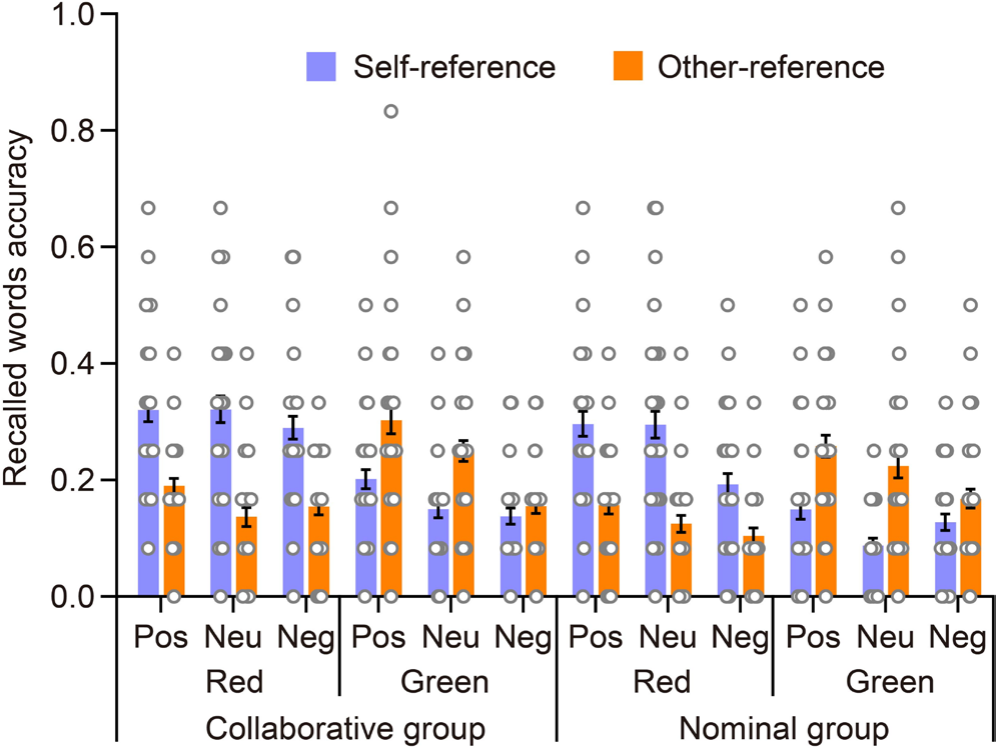
The accuracy for item memory during Recall 2. The figure portrays the data as a function of the encoding task, categorized by group, color, and emotional valence. The scattered dots depict the data for each individual, with error bars denoting standard errors. Pos, positive; Neu, neutral; Neg, negative.

Figure 6 illustrates the accuracy of item memory during Recall 2. The figure portrays the data as a function of the encoding task, categorized by group, color, and emotional valence. Accuracy for item memory of Recall 2 can also be found in Table 3. To explore the significance of the self‐reference effect in item memory of Recall 2, a mixed four‐way ANOVA was conducted. The variables were the same as for Recall 1.

**TABLE 3:** Accuracy (*M* and SE) for item memory of Recall 2, showing the function of color by encoding task by emotional valence by the group.

Color	Encoding task	Emotional valence	Group	*M*	SE
Red	Self‐reference	Positive	Collaborative	0.320	0.019
			Nominal	0.297	0.022
		Neutral	Collaborative	0.322	0.023
			Nominal	0.295	0.023
		Negative	Collaborative	0.290	0.020
			Nominal	0.193	0.018
	Other reference	Positive	Collaborative	0.190	0.013
			Nominal	0.157	0.015
		Neutral	Collaborative	0.137	0.016
			Nominal	0.125	0.015
		Negative	Collaborative	0.155	0.014
			Nominal	0.105	0.013
Green	Self‐reference	Positive	Collaborative	0.202	0.017
			Nominal	0.150	0.017
		Neutral	Collaborative	0.150	0.014
			Nominal	0.088	0.012
		Negative	Collaborative	0.138	0.014
			Nominal	0.128	0.014
	Other reference	Positive	Collaborative	0.303	0.024
			Nominal	0.258	0.019
		Neutral	Collaborative	0.250	0.018
			Nominal	0.225	0.021
		Negative	Collaborative	0.155	0.012
			Nominal	0.168	0.016

The ANOVA revealed a significant main effect of the encoding task, *F*(1,98) = 17.625, *p* < 0.001, $\eta_{p}^{2}=0.152$. Specifically, the accuracy of the self‐reference encoding task (*M* = 0.214, SE = 0.006) was significantly higher compared to that of the other‐reference encoding task (*M* = 0.186, SE = 0.005), providing evidence of the self‐reference effect. Furthermore, encoding task did not interact with group, *F*(1,98) = 2.071, *p* = 0.153, $\eta_{p}^{2}=0.021$. However, encoding task did significantly interact with color, *F*(1,98) = 355.715, *p* < 0.001, $\eta_{p}^{2}=0.784$. Post hoc tests showed a reliable self‐reference effect for words studied in red, *t*(98) = 15.563, *p* < 0.001, Cohen's *d* = 1.155, 95% CI (0.117, 0.166). For those studied in green, the pattern was reversed, *t*(98) = −9.236, *p* < 0.001, Cohen's *d* = −0.685, 95% CI (−0.108, −0.060). Encoding task did not interact with emotional valence, *F*(2,196) = 1.810, *p* = 0.166, $\eta_{p}^{2}=0.018$; nor did the interaction among encoding task, group, and emotional valence, *F*(2,196) = 0.918, *p* = 0.401, $\eta_{p}^{2}=0.009$.

However, the three‐way interaction among encoding task, color, and emotional valence was significant, as indicated by *F*(2,196) = 10.290, *p* < 0.001,$\eta_{p}^{2}=0.095$. Post hoc tests indicated that for words studied in red, all three types of emotional valences observed reliable self‐reference effects, with positive had a *t*(98) = 8.551, *p* < 0.001, Cohen's *d* = 1.102, 95% CI (0.082, 0.188); neutral: *t*(98) = 11.247, *p* < 0.001, Cohen's *d* = 1.450, 95% CI (0.124, 0.231); negative: *t*(98) = 7.076, *p* < 0.001, Cohen's *d* = 0.912, 95% CI (0.058, 0.165). In contrast, for words studied in green, reversed self‐reference effects were observed for positive and neutral words, with *t*(98) = −6.654, *p* < 0.001, Cohen's *d* = −0.858, 95% CI (−0.158, −0.052), and *t*(98) = −7.496, *p* < 0.001, Cohen's *d* = −0.966, 95% CI (−0.172, −0.065). Furthermore, the effect for negative valence was not reliable, *t*(98) = −1.799, *p* = 1.000, Cohen's *d* = −0.232, 95% CI (−0.082, 0.025). Additionally, the four‐way interaction among encoding task, group, color, and emotional valence was not found to be significant, *F*(2,196) = 0.254, *p* = 0.776, $\eta_{p}^{2}=0.003$.

In summary, a reliable self‐reference effect was observed in the item memory task of Recall 2, and this effect was not affected by whether the participants had previously collaborated. The self‐reference effect was found to be influenced by both the color and emotional valence of words. Specifically, when words were studied in red, the typical self‐reference effect was present across words with different emotional valences. However, for words studied in green, the self‐reference effect was reversed and only evident in positive and neutral words.

**FIGURE 7:**
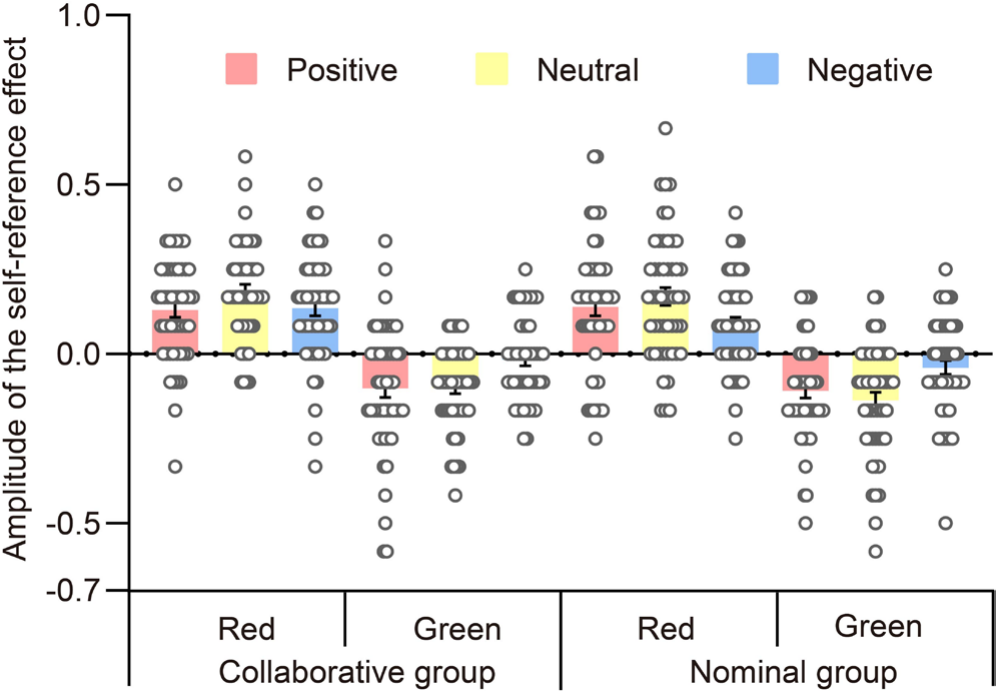
The amplitude of the self‐reference effect of Recall 2, which is demonstrated through the depiction of data by group, color, and emotional valence. Each scattered dot represents the data per individual, with error bars indicating standard errors.

Due to the impact of the emotional valence of words on the self‐reference effect related to Recall 2, we investigated a study to determine the magnitude of this effect and verify the presence of a self‐positivity bias. The mixed ANOVA incorporated group, color, and emotional valence as variables. For more information on the magnitude of this effect, see Figure 7.

The ANOVA did not confirm a main effect of emotional valence, *F*(2,196) = 1.810, *p* = 0.166, $\eta_{p}^{2}=0.003$, nor did it demonstrate a significant interaction between emotional valence and group, *F*(2,196) = 0.918, *p* = 0.401, $\eta_{p}^{2}=0.002$. However, emotional valence interacted with color, *F*(2,196) = 10.290, *p* < 0.001, $\eta_{p}^{2}=0.027$. Post hoc tests revealed that, in the case of green words, the magnitude of the self‐reference effect was significantly higher for positive words than for negative words, *t*(98) = −3.428, *p* = 0.010, Cohen's *d* = −0.486, 95% CI (−0.143, −0.011), and also for neutral words than for negative words, *t*(98) = −4.022, *p* = 0.001, Cohen's *d* = −0.570, 95% CI (−0.156, −0.024). However, positive words acted similarly to neutral words, *t*(98) = 0.594, *p* = 1.000, Cohen's *d* = 0.084, 95% CI (−0.053, 0.079). The magnitude of the self‐reference effect for red words was similar across all three emotional valences, with *p* ≥ 0.051. Additionally, the three‐way interaction of color, emotional valence, and group was not found to be significant, *F*(2,196) = 0.254, *p* = 0.776, $\eta_{p}^{2}=6.651\times 10^{-4}$. Thus, during the item memory task of Recall 2, a reversed self‐positivity bias was observed for words that had been studied in green, regardless of social collaboration.

#### 3.2.2 The Effects of Retrieving Sources During Recall 2

**FIGURE 8:**
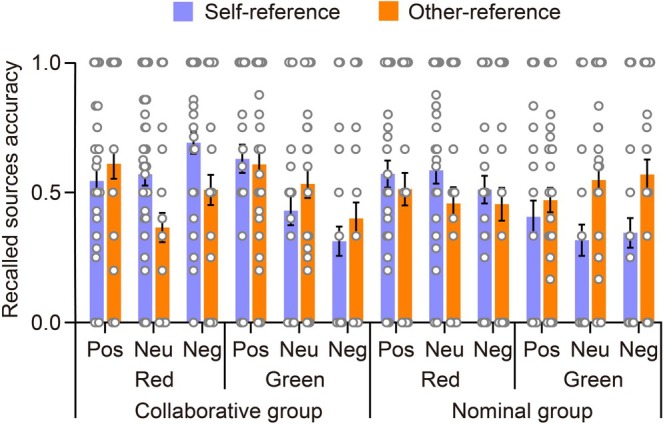
The CSIM rates in retrieving sources during Recall 2. The figure displays the data as a function of the encoding task, categorized by group, color, and emotional valence. The scattered dots depict the data for each individual, while the error bars denote standard errors. Pos, positive; Neu, neutral; Neg, negative.

Figure 8 illustrates the CSIM rates in retrieving sources during Recall 2. The figure displays the data as a function of the encoding task, categorized by group, color, and emotional valence. CSIM rates for source memory of Recall 2 can also be seen in Table 4. To explore the significance of the self‐reference effect in retrieving sources during Recall 2, the CSIM rates underwent a mixed four‐way ANOVA as that used in the above analyses of Recall 1.

**TABLE 4:** CSIM rates (*M* and SE) for source memory of Recall 2, showing the function of color by encoding task by emotional valence by the group.

Color	Encoding task	Emotional valence	Group	*M*	SE
Red	Self‐reference	Positive	Collaborative	0.545	0.044
			Nominal	0.572	0.052
		Neutral	Collaborative	0.570	0.043
			Nominal	0.586	0.051
		Negative	Collaborative	0.692	0.042
			Nominal	0.512	0.053
	Other reference	Positive	Collaborative	0.611	0.058
			Nominal	0.513	0.063
		Neutral	Collaborative	0.365	0.056
			Nominal	0.458	0.062
		Negative	Collaborative	0.510	0.058
			Nominal	0.455	0.063
Green	Self‐reference	Positive	Collaborative	0.630	0.055
			Nominal	0.407	0.062
		Neutral	Collaborative	0.430	0.056
			Nominal	0.317	0.060
		Negative	Collaborative	0.313	0.056
			Nominal	0.345	0.057
	Other reference	Positive	Collaborative	0.608	0.046
			Nominal	0.471	0.047
		Neutral	Collaborative	0.534	0.054
			Nominal	0.548	0.053
		Negative	Collaborative	0.400	0.061
			Nominal	0.569	0.057

The ANOVA did not reveal a significant main effect of the encoding task, *F*(1,98) = 0.203, *p* = 0.653 $\eta_{p}^{2}=0.002$. It was observed that the CSIM rates for the self‐reference encoding task (*M* = 0.493, SE = 0.016) were similar to those for the other‐reference encoding task (*M* = 0.504, SE = 0.017). Furthermore, encoding task did not interact with group, *F*(1,98) = 2.367, *p* = 0.127, $\eta_{p}^{2}=0.024$. However, encoding task did interact with color, *F*(1,98) = 22.598, *p* < 0.001, $\eta_{p}^{2}=0.187$. Post hoc tests revealed evidence of the self‐reference effect for words studied in red, with *t*(98) = 2.934, *p* = 0.022, Cohen's *d* = 0.242, 95% CI (0.009, 0.179); while this effect was reversed for words studied in green, with *t*(98) = −3.589, *p* = 0.003, Cohen's *d* = −0.296, 95% CI (−0.200, −0.030). The encoding task did not interact with emotional valence, *F*(2,196) = 0.065, *p* = 0.937, $\eta_{p}^{2}=6.649\times 10^{-4}$; nor did the three‐way interaction among encoding task, group, and emotional valence, *F*(2,196) = 1.332, *p* = 0.266, $\eta_{p}^{2}=0.013$.

However, the three‐way interaction of encoding task, color, and emotional valence was found to be statistically significant, *F*(2,196) = 6.289, *p* = 0.002, $\eta_{p}^{2}=0.060$. Post hoc tests did not find any evidence of the self‐reference effect, *p* ≥ 0.066. Additionally, the four‐way interaction of encoding task, group, color, and emotional valence was not found to be significant, *F*(2,196) = 0.607, *p* = 0.546, $\eta_{p}^{2}=0.006$.

Therefore, in the source memory task of Recall 2, a reliable self‐reference effect was observed for words that were studied in red, while it was reversed for words studied in green. This effect remained unaffected by whether participants had collaborated previously or not. The emotional valence of words did not play a role in determining this outcome.

## 4 Discussion

The current study intended to determine whether the self‐reference effect and self‐positivity bias manifest during collaboration and post‐collaboration sessions. Additionally, the study sought to assess whether these effects differ across various memory tasks. We have uncovered several key findings related to these effects, which we would like to discuss.

### 4.1 The Self‐Reference Effect Occurs in a Social Collaboration Setting, Regardless of Whether the Participants Have Collaborated

As anticipated, we observed a strong self‐reference effect in item memory during Recall 1, with words studied using self‐reference encoding performing better than those studied using other‐reference encoding. Therefore, we innovatively observed the self‐reference effect in a social collaborative setting, confirming our Hypothesis 1. This aligns with earlier studies on item memory tasks of individual memory (e.g., Clarkson et al. 2022; Kim et al. 2022; Rowell and Jaswal 2021; Yamawaki et al. 2017). Thus, the theories that describe the self‐reference effect, such as the working self, the self‐knowledge framework, and/or the automatic attentional account, are relevant to our current experiment.

Similar to past studies (e.g., Kalenzaga and Jouhaud 2018), the working self plays a crucial role in our current research by guiding individuals on which information to store and which experiences to recall. The self‐reference effect in our study can be further understood through the self‐knowledge framework (Hutchison et al. 2021; Kim et al. 2022; Nie, Zhou, et al. 2023; Rowell and Jaswal 2021). This means that activating pre‐existing self‐knowledge structures helps to organize and enhance the encoding and storage of self‐relevant information, resulting in the self‐reference effect. According to the automatic attentional account (Humphreys and Siu 2016; Hutchison et al. 2021; Rowell and Jaswal 2021; Sui and Humphreys 2017), automatic attentional responses to self‐cues assist in prioritizing the processing of current self‐relevant materials.

The self‐reference effect was consistently observed regardless of whether participants collaborated because it was not influenced by the group variable. This implies that the presence of the self‐reference effect was not affected by whether participants were in collaborative or nominal groups, indicating that the RSDH did not impact this effect. More precisely, the RSDH suggests that individuals' existing cognitive structure can lead to idiosyncratic cognitive organization for the information they learn, and preferred retrieval strategies may vary among collaborators, potentially causing disruptions in planned strategies (Guazzini et al. 2020; Maswood et al. 2022; Nie, Ke, et al. 2023; Nie and Liu 2024; Rossi‐Arnaud et al. 2020; also see Marion and Thorley 2016, for a review). Nevertheless, there is no indication that collaboration influences the self‐reference effect, indicating that the strategy disruption for recalling words from self‐reference and other‐reference encoding tasks is comparable.

Apart from the Recall 1 session, we also observed a significant self‐reference effect in item memory during Recall 2. This indicates that we were able to identify this effect in both the collaborative task and after the collaboration had ended, showcasing our innovation. Therefore, our Hypothesis 5 has been verified. Two implications emerge from this analysis. First, the importance of the working self, self‐knowledge framework, and/or automatic attentional account remains significant, regardless of whether the collaboration is ongoing or not. Second, the comparability of strategy disruption for words studied by self‐reference and other‐reference encoding tasks is evident in both the collaboration and post‐collaboration sessions.

Nonetheless, the above findings suggest that the benefit of recalling self‐reference encoded information, known as the self‐reference effect, remains relatively constant. The pattern of self‐reference does not vary depending on the method of retrieving information, whether individual or collaborative. This implies that if one wishes to recall more self‐reference encoded information, collaborating with others may not be the best choice. However, the current study is the first attempt to incorporate self‐reference in social collaboration. The lack of susceptibility of the self‐reference effect to collaboration still needs to be further verified.

### 4.2 The Self‐Reference Effect Varies Depending on Whether the Words Were Studied in Red or Green

Although the self‐reference effect was not influenced by the collaborative manipulation, we observed that the pattern of this effect was determined by the encoding color—either red or green—for words. Specifically, only the words studied in red showed the typical self‐reference effect, while the pattern for green words was reversed. This verifies our Hypothesis 3 and Hypothesis 4. On the one hand, this implies that mechanisms related to the theories, such as the working self (Kalenzaga and Jouhaud 2018), the self‐knowledge framework (Hutchison et al. 2021; Kim et al. 2022; Nie, Zhou, et al. 2023; Rowell and Jaswal 2021), and the automatic attentional account (Hutchison et al. 2021; Rowell and Jaswal 2021; Sui and Humphreys 2017), apply to words studied in red.

Our current study is the first attempt to differentiate the role of different colors in episodic memory. Previous research had combined them (Nie and Deng 2023; Nie et al. 2019; Nie, Ke, et al. 2023; Nie, Li, et al. 2023), even though studies that focused on the self‐reference effect (e.g., Nie, Zhou, et al. 2023). The finding that red and green behave differently is a novel discovery in our current research. Strikingly, we found this discovery in both item recall and source memory. This similarity can be attributed to the reliance on the recollection process in both tasks, as indicated by the dual‐process theories (Minor and Herzmann 2019; Nie, Zhou, et al. 2023; Symeonidou and Kuhlmann 2022).

The difference between the red and green scenarios could be attributed to four potential causes. The first possible cause could be participants' process bias, while the others could be attributed to the nature of colors. The first potential cause is that participants may have consistently processed different colors in varying ways, with one color affecting their perception more than the other, such as red over green. If these distinctions were examined in previous studies (e.g., Nie and Deng 2023; Nie et al. 2019; Nie, Ke, et al. 2023; Nie, Li, et al. 2023), a comparable pattern might emerge.

The second possibility is that the trait adjectives are more closely linked to people, leading them to associate warm colors like vibrant red with these words rather than cooler tones like green. This ultimately results in participants associating self‐reference words with themselves. This discrepancy may not be present in studies that utilize common words, as shown in other research (e.g., Nie and Deng 2023; Nie et al. 2019; Nie, Ke, et al. 2023; Nie, Li, et al. 2023).

The third point to consider is the psychological differences between red and green. Red is commonly perceived as a color that can stimulate quick action and a sense of urgency in people. Studies have shown that seeing red can make individuals experience a greater sense of urgency and heightened anxiety levels (El Jouhri et al. 2023). Furthermore, red can enhance a person's physical and sexual attractiveness, but it may also have negative effects, such as athletes wearing red being more likely to underperform in competitions, and individuals exposed to a red environment before exams being more prone to making mistakes. On the other hand, green brings a sense of peace and tranquility, helping to alleviate anxiety and promote a sense of calmness. These contrasting characteristics drew more attention from our participants to the red case as opposed to the green case.

The fourth account may be based on the idea that red holds rich connotations and symbolic meanings in Chinese culture. In China, red symbolizes joy and auspiciousness, representing passion and vitality, giving people a sense of energy and vigor. Historically, red is closely tied to revolution and progress. Red plays a significant role in various traditions and customs, such as pasting red couplets during the Spring Festival, brides wearing red attire at weddings, and individuals wearing red clothing and tying red belts during their zodiac year. These customs emphasize the importance of red in Chinese culture. These specific connotations and symbolic meanings may cause our current participants to pay more attention to red stimuli, thereby enhancing their memory performance.

The self‐reference effect was observed to be independent of social collaboration. This statement contradicts our further differentiation in both Hypothesis 1 and Hypothesis 5. Similarly, the influence of color on this effect remained unaffected by social collaboration. Both the typical self‐reference effect seen with red words and the opposite trend in green words were comparable across both collaborative and nominal groups. The findings were not only revealed during the ongoing collaboration of Recall 1 but also persisted during the post‐collaboration session of Recall 2. Thus, the mechanisms raised by RSDH (Abel and Bäuml 2020; Nie and Deng 2023; Nie et al. 2019; Nie and Guo 2023; Nie, Li, et al. 2023; also see Marion and Thorley 2016, for a review) do not affect how color influences the self‐reference effect.

The findings above provide us with at least two important implications. First, it appears that the retention of contextual information from self‐reference encoded events varies depending on the source—red or green. To improve memory performance for self‐reference encoded events, it may be more effective to place them in a red context rather than in a green one. Second, the advantage of recalling information in a red context does not depend on whether one is engaging in social collaboration or not. In other words, remembering oneself or remembering with others seems to yield similar results. This suggests that if one wants to recall more contextual information related to self‐reference encoded events, collaborating with others may not be the most effective choice.

### 4.3 The Self‐Positivity Bias Is Evident in Both Item Memory and Source Memory Tasks

Different from the role of social collaboration, we discovered that the self‐reference effect was influenced by the emotional valence of words in both item memory and source memory tasks. However, the influence differed between the two tasks.

In both the Recall 1 and Recall 2 sessions, it was observed that the amplitude of the self‐reference effect was significantly greater for positive words compared to negative words. This highlights the presence of a self‐positivity bias. Therefore, our Hypothesis 2 and Hypothesis 6 are verified. This finding aligns with some previous studies (Arnaud et al. 2005; Kalenzaga and Jouhaud 2018), but is inconsistent with some other investigations (Grilli et al. 2018; Kim et al. 2022). The working self, self‐knowledge framework, and automatic attentional account are likely to have a stronger impact on current positive words compared to negative words.

The presence of self‐positivity bias was found regardless of whether participants were in a social collaboration setting, as the group did not interact with emotional valence. These findings contradict our further differentiation in Hypothesis 2 and Hypothesis 4. This suggests that the mechanisms of RSDH (Abel and Bäuml 2020; Nie and Deng 2023; Nie et al. 2019; Nie and Guo 2023; Nie, Li, et al. 2023; also see Marion and Thorley 2016, for a review) are not connected to the self‐positivity bias. Furthermore, the self‐positivity bias was found exclusively with words encoded in green, but not in red. This highlights that the contrast between red and green colors can have an impact on the self‐reference effect and self‐positivity bias. Further research is required to explore this phenomenon in more depth in the future.

In terms of the source memory of Recall 1, only the positive words showed a self‐reference effect, while neutral and negative words did not. This indicates a self‐positivity bias in the source memory task. Kim et al. (2022) and Nie, Zhou, et al. (2023) have both confirmed the self‐positivity bias in source memory. It is worth noting that while the current study focused on retrieving self‐reference or other‐reference encoding tasks for words, Kim et al. (2022) had participants retrieve the emotional sources for words, while Nie, Zhou, et al. (2023) had participants retrieve the encoding color for words. The demonstration shows that the type of source is not the main factor in determining the self‐positivity bias. In contrast, we did not find evidence of the self‐positivity bias in source memory during Recall 2, indicating that the effect did not persist after collaboration. This contradicts Hypothesis 7 and Hypothesis 8.

The findings above offer at least two important implications. First, it seems that retaining self‐reference encoded events is more effective for positive events than for negative or neutral ones. To enhance memory performance for self‐reference encoded events, focusing on positive events may yield better results. However, the color context in which events are placed, green but not red, does have an impact. Second, the advantage of recalling information about positive events is not influenced by whether a person is engaging in social collaboration. In other words, remembering information about oneself or remembering with others produces similar outcomes. This suggests that if one desires to retrieve more positive information related to self‐reference situations, collaborating with others may not be the most effective option.

## 5 Limitations and Future Directions

The current experiment has also uncovered certain constraints, prompting us to propose some directions for future research.

First, the encoding task needs to be considered. Our current study utilized the standard paradigm to investigate the self‐reference effect. Participants engaged in self‐encoding tasks as opposed to other‐encoding tasks. This paradigm has been confirmed to easily evoke a significant self‐reference effect (Clarkson et al. 2022; Howell and Zelenski 2017; Kim et al. 2022; Rowell and Jaswal 2021). Furthermore, research has shown that the encoding strategy used plays a vital role in eliciting this effect (Gutchess et al. 2015). It is recommended that future studies incorporate a variety of encoding strategies to further investigate the alterations in both the self‐reference effect and self‐positivity bias.

Second, we should consider the collaboration procedure, including the possibility of incorporating turn‐taking. Besides, in the current study, the free‐flowing procedure was used. Previous research has indicated that turn‐taking can improve performance inhibition compared to free‐flowing collaboration. This is because participants' retrieval strategies are more vulnerable to disruption in the former scenario (Nie and Guo 2023, 2024; also see Marion and Thorley 2016, for a meta‐analysis). It is uncertain whether the advantage induced by self‐encoding can still be observed when turn‐taking is implemented, as well as whether the self‐positive bias will remain evident. Future studies are strongly encouraged to investigate how various collaborative procedures impact episodic memory for stimuli encoded through self‐encoding, as well as the influence of the differing emotional valence of the stimuli.

Third, the characteristics of the participants should be considered. As previously mentioned, researchers have investigated the reliability of both the self‐reference effect and the self‐positivity bias in individuals of different age groups and with various health conditions. The appearance and amplitude of these effects show distinct patterns that correspond to each other (Grilli et al. 2018; Jackson et al. 2019; Moses‐Payne et al. 2022). However, as our study is the first attempt to examine the self‐reference effect and self‐positivity bias in social interaction scenarios, future research needs to concentrate on analyzing how participants' characteristics, such as personality, age, physical and mental health, and social relationships between collaborators, influence these effects.

Fourth, when investigating source memory, it is important to consider the choice of paradigm. From our understanding, three paradigms can be utilized: sequential paradigm, exclusion paradigm, and three‐key paradigm (Nie et al. 2011; Nie, Ke, et al. 2023; Nie, Li, et al. 2023). In our current study, we employed the sequential paradigm in which responses for item memory and source memory were made in succession. In the three‐key paradigm, participants are required to provide three separate responses: one for items studied from Source A, one for items from Source B, and another for new items. Through this paradigm, we can compare the two memory tasks as their responses are given simultaneously. In the exclusion paradigm, participants are tasked with distinguishing between items learned from Source A and other items. Therefore, it would be valuable to investigate whether the self‐reference effect and self‐positivity bias occur within the three‐key paradigm or the exclusion paradigm. This avenue may present a promising direction for further research.

Fifth, the number of collaborators should be taken into consideration. The current study only involved two participants during the collaborative phase, making it uncertain whether similar data patterns would emerge with more collaborators involved. A meta‐analysis review has shown that the presence of more people can greatly interfere with retrieval strategies during collaboration (refer to Marion and Thorley 2016, for a review). This raises the question of whether our findings are also influenced by the size of the collaborators.

Last, when considering the valence and arousal of the affective words used in our study, we were unable to control the arousal level due to the limited number of word options available. Consequently, we only included positive, neutral, and negative words, while neglecting the arousal level. Therefore, we are unable to determine whether the emotion‐related effects observed in the current study are a result of arousal, valence, or both. The relationship between valence and arousal has long been a contentious topic that has been extensively explored by many researchers. Numerous theoretical models have been developed to elucidate the variations in memory retention for arousing events compared to other events (for a comprehensive review, see Bowen et al. 2018). Therefore, to gather supporting evidence on whether emotional arousal and valence manifest distinct characteristics in social collaborative situations, it is advisable to consider both factors.

## 6 Conclusion

We have innovatively expanded upon the self‐reference effect and the self‐positivity bias in a social collaborative setting. Specifically, (i) the self‐reference effect for item memory was present in both ongoing and enduring collaborative sessions for words studied in red, while it was reversed for those studied in green. Therefore, the mechanisms of the working self, self‐knowledge framework, and automatic attentional account are involved during collaborative sessions. The contribution may vary depending on the encoding color of words. (ii) During the ongoing collaborative session, there was an effect of self‐positivity bias in source memory. A reversed self‐positivity bias was observed in item memory for words that had been studied in green. This suggests that our knowledge of positive self‐elements is more readily activated during the session, garnering greater automatic attention compared to negative aspects. (iii) Whether participants collaborated or not did not impact the effects we were studying. This suggests that the theories contributing to these effects are not sensitive to the social collaboration between participants.

## Ethics Statement

All procedures followed the ethical standards of the responsible committee on human experimentation (institutional and national) and the guidelines of the Helsinki Declaration. The Science and Technology Ethics Committee of Shanxi Normal University approved all protocols.

## Consent

Informed consent was obtained from all individual participants included in the experiments.

## Conflicts of Interest

The authors declare no conflicts of interest.

## Data Availability

The datasets generated and/or analyzed and the materials of the current study will be available from the corresponding author on request. The related information can also be accessed at https://osf.io/ue8x3/?view_only=52108c24439a43ef8ab7b726f00214a6.

## References

[pchj70006-bib-0001] Abel, M. , and K. T. Bäuml . 2020. “Social Interactions Can Simultaneously Enhance and Distort Memories: Evidence From a Collaborative Recognition Task.” Cognition 200: 104254. 10.1016/j.cognition.2020.104254. 32192980

[pchj70006-bib-0002] Arnaud, D. , C. Christine , and V. D. L. Martial . 2005. “Affective Valence and the Self‐Reference Effect: Influence of Retrieval Conditions.” British Journal of Psychology 96, no. 4: 457–466. 10.1348/000712605X53218. 16248936

[pchj70006-bib-0003] Bärthel, G. A. , I. Wessel , R. J. Huntjens , and J. Verwoerd . 2017. “Collaboration Enhances Later Individual Memory for Emotional Material.” Memory 25, no. 5: 636–646. 10.1080/09658211.2016.1208248. 27403926

[pchj70006-bib-0004] Basden, B. H. , D. R. Basden , S. Bryner , and R. L. Thomas . 1997. “A Comparison of Group and Individual Remembering: Does Collaboration Disrupt Retrieval Strategies?” Journal of Experimental Psychology: Learning, Memory, and Cognition 23: 1176–1189. 10.1037/0278-7393.23.5.1176. 9293628

[pchj70006-bib-0005] Bell, R. , L. Mieth , and A. Buchner . 2017. “Emotional Memory: No Source Memory Without Old–New Recognition.” Emotion 17, no. 1: 120–130. 10.1037/emo0000211. 27504597

[pchj70006-bib-0006] Bowen, H. J. , S. M. Kark , and E. A. Kensinger . 2018. “NEVER Forget: Negative Emotional Valence Enhances Recapitulation.” Psychonomic Bulletin & Review 25, no. 3: 870–891. 10.3758/s13423-017-1313-9. 28695528 PMC6613951

[pchj70006-bib-0007] Clarkson, T. R. , S. J. Cunningham , C. Haslam , and A. Kritikos . 2022. “Is Self Always Prioritised? Attenuating the Ownership Self‐Reference Effect in Memory.” Consciousness and Cognition 106: 103420. 10.1016/j.concog.2022.103420. 36274390

[pchj70006-bib-0008] Durbin, K. A. , K. J. Mitchell , and M. K. Johnson . 2017. “Source Memory That Encoding Was Self‐Referential: The Influence of Stimulus Characteristics.” Memory 25, no. 9: 1191–1200. 10.1080/09658211.2017.1282517. 28276984 PMC5548658

[pchj70006-bib-0009] El Jouhri, A. , A. El Sharkawy , H. Paksoy , et al. 2023. “The Influence of a Color Themed HMI on Trust and Take‐Over Performance in Automated Vehicles.” Frontiers in Psychology 13, no. 14: 1128285. 10.3389/fpsyg.2023.1128285. PMC10382069 37519355

[pchj70006-bib-0010] Faul, F. , E. Erdfelder , A. Buchner , and A. G. Lang . 2009. “Statistical Power Analyses Using G*Power 3.1: Tests for Correlation and Regression Analyses.” Behavior Research Methods 41, no. 4: 1149–1160. 10.3758/BRM.41.4.1149. 19897823

[pchj70006-bib-0011] Greeley, G. D. , V. Chan , H. Choi , and S. Rajaram . 2024. “Collaborative Recall and the Construction of Collective Memory Organization: The Impact of Group Structure.” Topics in Cognitive Science 16, no. 2: 282–301. 10.1111/tops.12639. 36780338

[pchj70006-bib-0012] Grilli, M. D. , C. B. Woolverton , M. Crawford , and E. L. Glisky . 2018. “Self‐Reference and Emotional Memory Effects in Older Adults at Increased Genetic Risk of Alzheimer's Disease.” Aging, Neuropsychology, and Cognition 25, no. 2: 186–199. 10.1080/13825585.2016.1275508. 28044474

[pchj70006-bib-0013] Grysman, A. , C. B. Harris , A. J. Barnier , and G. Savage . 2020. “Long‐Married Couples Recall Their Wedding Day: The Influence of Collaboration and Gender on Autobiographical Memory Recall.” Memory 28, no. 1: 18–33. 10.1080/09658211.2019.1673428. 31615338

[pchj70006-bib-0014] Guazzini, A. , E. Guidi , C. Cecchini , and E. Yoneki . 2020. “Collaborative Facilitation and Collaborative Inhibition in Virtual Environments.” Future Internet 12, no. 7: 118–137. 10.3390/fi12070118.

[pchj70006-bib-0015] Gutchess, A. , and E. A. Kensinger . 2018. “Shared Mechanisms May Support Mnemonic Benefits From Self‐Referencing and Emotion.” Trends in Cognitive Sciences 22, no. 8: 712–724. 10.1016/j.tics.2018.05.001. 29886010 PMC6652178

[pchj70006-bib-0016] Gutchess, A. H. , R. Sokal , J. A. Coleman , G. Gotthilf , L. Grewal , and N. Rosa . 2015. “Age Differences in Self‐Referencing: Evidence for Common and Distinct Encoding Strategies.” Brain Research 1612: 118–127. 10.1016/j.brainres.2014.08.033. 25223905 PMC4362921

[pchj70006-bib-0017] Harris, C. B. , A. J. Barnier , J. Sutton , and G. Savage . 2019. “Features of Successful and Unsuccessful Collaborative Memory Conversations in Long‐Married Couples.” Topics in Cognitive Science 11, no. 4: 668–686. 10.1111/tops.12350. 29851268

[pchj70006-bib-0018] Harris, C. B. , P. Van Bergen , S. A. Harris , N. McIlwain , and A. Arguel . 2022. “Here's Looking at You: Eye Gaze and Collaborative Recall.” Psychological Research 86, no. 3: 769–779. 10.1007/s00426-021-01533-2. 34095971

[pchj70006-bib-0019] Hood, A. V. B. , S. R. Whillock , M. L. Meade , and K. A. Hutchison . 2023. “Does Collaboration Help or Hurt Recall? The Answer Depends on Working Memory Capacity.” Journal of Experimental Psychology: Learning, Memory, and Cognition 49, no. 3: 350–370. 10.1037/xlm0001155. 36006719

[pchj70006-bib-0020] Howell, G. T. , and J. M. Zelenski . 2017. “Personality Self‐Concept Affects Processing of Trait Adjectives in the Self‐Reference Memory Paradigm.” Journal of Research in Personality 66: 1–13. 10.1016/j.jrp.2016.12.001.

[pchj70006-bib-0021] Humphreys, G. W. , and J. Siu . 2016. “Attentional Control and the Self: The Self‐Attention Network (SAN).” Cognitive Neuroscience 7: 5–17. 10.1080/17588928.2015.1044427. 25945926

[pchj70006-bib-0022] Hutchison, J. , J. Ross , and S. J. Cunningham . 2021. “Development of Evaluative and Incidental Self‐Reference Effects in Childhood.” Journal of Experimental Child Psychology 210: 105197. 10.1016/j.jecp.2021.105197. 34090236

[pchj70006-bib-0023] Jackson, J. D. , C. Luu , A. Vigderman , E. D. Leshikar , and A. Gutchess . 2019. “Reduction of the Self‐Reference Effect in Younger and Older Adults.” Psychology & Neuroscience 12, no. 2: 257–270. 10.1037/pne0000142. 31263517 PMC6602554

[pchj70006-bib-0024] JASP Team . 2023. “JASP.” Version 0.18.3 [Computer Software].

[pchj70006-bib-0025] Kalenzaga, S. , and V. Jouhaud . 2018. “The Self‐Reference Effect in Memory: An Implicit Way to Assess Affective Self‐Representations in Social Anxiety.” Memory 26, no. 7–8: 894–903. 10.1080/09658211.2018.1430833. 29378468

[pchj70006-bib-0026] Ke, C. , A. Nie , and R. Zhang . 2017. “The Modulation of Recall Task on Collaborative Inhibition and Error Pruning: The Influence of Emotional Valence and Level of Processing.” Acta Psychologica Sinica 49, no. 6: 733–744. 10.3724/SP.J.1041.2017.00733.

[pchj70006-bib-0027] Kim, K. , A. M. Banquer , S. N. Resnik , J. D. Johnson , and L. Fernandez . 2022. “Self‐Reference and Cognitive Effort: Source Memory for Affectively Neutral Information Is Impaired Following Negative Compared to Positive Self‐Referential Processing.” Journal of Cognitive Psychology 34, no. 7: 833–845. 10.1080/20445911.2022.2067553.

[pchj70006-bib-0028] Li, M. , and A. Nie . 2023. “Discrepancies in Episodic Memory: Different Patterns of Age Stereotypes in Item and Source Memory.” Current Psychology 42: 5873–5885. 10.1007/s12144-021-01937-8.

[pchj70006-bib-0029] Marion, S. B. , and C. Thorley . 2016. “A Meta‐Analytic Review of Collaborative Inhibition and Post‐Collaborative Memory: Testing the Predictions of the Retrieval Strategy Disruption Hypothesis.” Psychological Bulletin 142, no. 11: 1141–1164. 10.1037/bul0000071. 27618544

[pchj70006-bib-0030] Maswood, R. , C. C. Luhmann , and S. Rajaram . 2022. “Persistence of False Memories and Emergence of Collective False Memory: Collaborative Recall of DRM Word Lists.” Memory 30, no. 4: 465–479. 10.1080/09658211.2021.1928222. 34037498

[pchj70006-bib-0031] Minor, G. , and G. Herzmann . 2019. “Effects of Negative Emotion on Neural Correlates of Item and Source Memory During Encoding and Retrieval.” Brain Research 1718: 32–45. 10.1016/j.brainres.2019.05.001. 31054883

[pchj70006-bib-0032] Montefinese, M. , E. Ambrosini , B. Fairfield , and N. Mammarella . 2014. “The Adaptation of the Affective Norms for English Words (ANEW) for Italian.” Behavior Research Methods 46, no. 3: 887–903. 10.3758/s13428-013-0405-3. 24150921

[pchj70006-bib-0033] Montoro‐Membila, N. , R. Maswood , B. Molina , S. Rajaram , and T. Bajo . 2022. “Neurocognitive Mechanisms of Collaborative Recall.” Neurobiology of Learning and Memory 193: 107639. 10.1016/j.nlm.2022.107639. 35598824

[pchj70006-bib-0034] Moses‐Payne, M. E. , G. Chierchia , and S. J. Blakemore . 2022. “Age‐Related Changes in the Impact of Valence on Self‐Referential Processing in Female Adolescents and Young Adults.” Cognitive Development 61: 101128. 10.1016/j.cogdev.2021.101128. PMC8791274 35125644

[pchj70006-bib-0035] Nie, A. , and C. Deng . 2023. “Detrimental and Beneficial Effects in Ongoing and Lasting Collaborative Memory: Insight From the Emotional Timeout Procedure.” Advances in Cognitive Psychology 19, no. 1: 59–79. 10.5709/acp-0376-4.

[pchj70006-bib-0036] Nie, A. , and B. Guo . 2023. “Benefits and Detriments of Social Collaborative Memory in Turn‐Taking and Directed Forgetting.” Perceptual and Motor Skills 130, no. 3: 1040–1076. 10.1177/00315125231163626. 36988341

[pchj70006-bib-0037] Nie, A. , and B. Guo . 2024. “Differentiating the DF Effect in Episodic Memory: Evaluating the Contribution of the Procedures of Collaborative Memory.” Journal of General Psychology 151, no. 3: 223–270. 10.1080/00221309.2023.2252133. 37671532

[pchj70006-bib-0038] Nie, A. , C. Guo , and M. Shen . 2011. “The Influence of the Testing Paradigm on Location Source Retrieval: An Event‐Related Potentials Study.” Acta Psychologica Sinica 43, no. 5: 473–482. 10.3724/SP.J.1041.2011.00473.

[pchj70006-bib-0039] Nie, A. , C. Ke , B. Guo , M. Li , and Y. Xiao . 2023. “Collaborative Memory for Categorized Lists: Ongoing and Lasting Effects Are Sensitive to Episodic Memory Tasks.” Current Psychology 42: 3870–3887. 10.1007/s12144-021-01684-w.

[pchj70006-bib-0040] Nie, A. , C. Ke , M. Li , and B. Guo . 2019. “Disrupters as Well as Monitors: Roles of Others During and After Collaborative Remembering in DRM Procedure.” Advances in Cognitive Psychology 15, no. 4: 276–289. 10.5709/acp-0275-1. 32494313 PMC7251628

[pchj70006-bib-0041] Nie, A. , and M. Li . 2021. “Professional Discrepancies of Doctors and Lawyers in Episodic Memory: Modulations of Professional Morality and Warning.” PsyCh Journal 10, no. 5: 707–731. 10.1002/pchj.457. 34137498

[pchj70006-bib-0042] Nie, A. , and M. Li . 2024. “Detriments and Benefits in Collaborative Memory of Social Information: Insight From Gender Stereotypes.” American Journal of Psychology 137, no. 1: 19–37.

[pchj70006-bib-0043] Nie, A. , M. Li , M. Li , Y. Xiao , and S. Wang . 2023. “Together We Lose or Gain: Ongoing and Enduring Impacts of Collaboration in Episodic Memory of Emotional DRM Lists.” Current Psychology 42: 27965–27982. 10.1007/s12144-022-03940-z.

[pchj70006-bib-0044] Nie, A. , M. Li , Q. Wang , and C. Zhang . 2024. “The Isolation Between Part‐Set Cues and Social Collaboration in Episodic Memory Is Dependent: Insight From Ongoing and Post‐Collaboration.” Scandinavian Journal of Psychology 65: 981–999. 10.1111/sjop.13042. 39513467

[pchj70006-bib-0045] Nie, A. , and S. Liu . 2024. “The Detrimental and Beneficial Effects of Collaboration Are Sensitive to Both Collaborative Frequency and Collaborative Order but Not to the Encoding Task.” Journal of General Psychology, Advance Online Publication. 10.1080/00221309.2024.2385098. 39074043

[pchj70006-bib-0046] Nie, A. , and Y. Wu . 2023. “Differentiation of the Contribution of Familiarity and Recollection to the Old/New Effects in Associative Recognition: Insight From Semantic Relation.” Brain Sciences 13: 553. 10.3390/brainsci13040553. 37190517 PMC10136778

[pchj70006-bib-0047] Nie, A. , W. Zhou , and Y. Xiao . 2023. “Sensitivity of Late ERP Old/New Effects in Source Memory to Self‐Referential Encoding Focus and Stimulus Emotionality.” Neurobiology of Learning and Memory 203: 107795. 10.1016/j.nlm.2023.107795. 37394031

[pchj70006-bib-0048] Pepe, N. W. , Q. Wang , and S. Rajaram . 2021. “Collaborative Remembering in Ethnically Uniform and Diverse Group Settings.” Journal of Applied Research in Memory and Cognition 10, no. 1: 95–103. 10.1016/j.jarmac.2020.08.001.

[pchj70006-bib-0049] Pereira, D. R. , A. C. Teixeira‐Santos , A. Sampaio , and A. P. Pinheiro . 2022. “Examining the Effects of Emotional Valence and Arousal on Source Memory: A Meta‐Analysis of Behavioral Evidence.” Emotion 23, no. 6: 1740–1763. 10.1037/emo0001188. 36480404

[pchj70006-bib-0050] Roca, P. , C. Vázquez , and D. Ondé . 2023. “The Hedonic and Arousal Affect Scale (HAAS): A Brief Adjective Checklist to Assess Affect States.” Personality and Individual Differences 207: 112151. 10.1016/j.paid.2023.112151.

[pchj70006-bib-0051] Rossi‐Arnaud, C. , P. Spataro , D. Bhatia , F. Doricchi , S. Mastroberardino , and V. Cestari . 2020. “Long‐Lasting Positive Effects of Collaborative Remembering on False Assents to Misleading Questions.” Acta Psychologica 203: 102986. 10.1016/j.actpsy.2019.102986. 31887634

[pchj70006-bib-0052] Rossi‐Arnaud, C. , P. Spataro , A. Santirocchi , M. C. Pesola , L. Costantini , and V. Cestari . 2023. “Positive and Negative Effects of Collaboration on Suggestibility and False Memory in Online Groups.” Current Psychology 24: 1–13. 10.1007/s12144-023-04775-y. PMC10208203 37359627

[pchj70006-bib-0053] Rowell, S. F. , and V. K. Jaswal . 2021. “I Remember Being Nice: Self‐Enhancement Memory Bias in Middle Childhood.” Memory 29, no. 2: 261–269. 10.1080/09658211.2021.1877307. 33507125

[pchj70006-bib-0054] Saraiva, M. , P. B. Albuquerque , and M. V. Garrido . 2023. “Collaborative Inhibition Effect: The Role of Memory Task and Retrieval Method.” Psychological Research 87, no. 8: 2548–2558. 10.1007/s00426-023-01821-z. 37027039

[pchj70006-bib-0055] Sui, J. , and G. W. Humphreys . 2017. “The Ubiquitous Self: What the Properties of Self‐Bias Tell Us About the Self.” Annals of the New York Academy of Sciences 1396: 222–235. 10.1111/nyas.13197. 27918835 PMC6029667

[pchj70006-bib-0056] Symeonidou, N. , and B. G. Kuhlmann . 2022. “Better Memory for Emotional Sources? A Systematic Evaluation of Source Valence and Arousal in Source Memory.” Cognition and Emotion 36, no. 2: 300–316. 10.1080/02699931.2021.2008323. 34843428

[pchj70006-bib-0057] Van Den Joke, B. , B. Leen , R. Gina , D. Eva , M. Kalina , and H. Joeri . 2014. “Factorial Validity of the Personality Adjective Checklist in a Dutch‐Speaking Sample.” Journal of Personality Assessment 96, no. 2: 245–251. 10.1080/00223891.2013.840306. 24117012

[pchj70006-bib-0058] Wang, Y. , L. Zhou , and Y. Luo . 2008. “The Pilot Establishment and Evaluation of Chinese Affective Words System.” Chinese Mental Health Journal 22, no. 8: 608–612. 10.3321/j.issn:10006729.2008.08.014.

[pchj70006-bib-0059] Warriner, A. B. , V. Kuperman , and M. Brysbaert . 2013. “Norms of Valence, Arousal, and Dominance for 13,915 English Lemmas.” Behavior Research Methods 45, no. 4: 1191–1207. 10.3758/s13428-012-0314-x. 23404613

[pchj70006-bib-0060] Xiao, Y. , and A. Nie . 2023. “Does Expecting Matter? The Impact of Experimentally Established Expectations on Subsequent Memory Retrieval of Emotional Words.” Journal of Intelligence 11: 130. 10.3390/jintelligence11070130. 37504773 PMC10381812

[pchj70006-bib-0061] Yamawaki, R. , K. Nakamura , T. Aso , Y. Shigemune , H. Fukuyama , and T. Tsukiura . 2017. “Remembering My Friends: Medial Prefrontal and Hippocampal Contributions to the Self‐Reference Effect on Face Memories in a Social Context.” Human Brain Mapping 38, no. 8: 4256–4269. 10.1002/hbm.23662. 28548263 PMC6867012

[pchj70006-bib-0062] Ye, J. , A. Nie , and S. Liu . 2019. “How Do Word Frequency and Memory Task Influence Directed Forgetting: An ERP Study.” International Journal of Psychophysiology 146: 157–172. 10.1016/j.ijpsycho.2019.10.005. 31655184

[pchj70006-bib-0063] Zhang, H. , Y. Liu , X. Wang , et al. 2023. “Benefits of Collaborative Remembering in Older and Younger Couples: The Role of Conversation Dynamics and Gender.” Memory 31, no. 3: 406–420. 10.1080/09658211.2023.2166963. 36651520

[pchj70006-bib-0064] Zhou, W. , A. Nie , Y. Xiao , S. Liu , and C. Deng . 2020. “Is Color Source Retrieval Sensitive to Emotion? Electrophysiological Evidence From Old/New Effects.” Acta Psychologica 210: 103156. 10.1016/j.actpsy.2020.103156. 32801072

